# Supplementary material for: Examining public support for Ukraine’s defense against autocratic aggression
Source: Nat Commun. 2026 Jan 9;17:414. doi: 10.1038/s41467-025-67913-z (PMC12796306; doi:10.1038/s41467-025-67913-z)
Supplement: Supplementary file 1 — Supplementary Information [file 41467_2025_67913_MOESM1_ESM.pdf]

Supplementary Information

Examining public support for Ukraine's defense against autocratic aggression

Lukas Rudolph[a], Fabian Haggerty[b], and Paul W. Thurner[c]

Nature Communications

Contents

1 Supplementary Discussion **S2**

1.1 Literature and Empirical Evidence on Public Opinion Regarding Ukraine Support S2

1.2 Comparison to the Pre-Registration S3

2 Supplementary Methods **S10**

2.1 Design of the Conjoint Experiment S10

2.2 Design of the Vignette Experiment S13

3 Supplementary Results **S15**

3.1 Additional Results for Conjoint Experiment S15

3.2 Additional Results for Vignette Experiment S21

3.3 Heterogeneity by Attitudes Towards War and Peace and Foreign Policy Values . . S24

3.4 Robustness Checks S26

3.5 Statistics Corresponding to Main Figures and Their Interpretation S33

3.6 Information on Dataset and Software S42

[a]University of Konstanz. lukas.rudolph@uni-konstanz.de
[b]LMU Munich. fabian.haggerty@gsi.lmu.de
[c]LMU Munich. paul.thurner@gsi.lmu.de

1 Supplementary Discussion

1.1 Literature and Empirical Evidence on Public Opinion Regarding Ukraine Support

After three years of extraordinary international financial and military support [see 1, for country-level figures], cracks in the backing of Ukraine show. Until 2024, the rhetoric of the ruling elites in the US and major European countries emphasized strong resolve "as long as it takes" (see e.g., President Biden in 2022 [2], repeated in 2023 [3] and 2024 [4], the German-Ukrainian bilateral 'Agreement on security cooperation and long-term support' [5] or the NATO-Ukraine Council statement in 2024 [6]; similarly, the EU parliament emphasized to "Provide Ukraine with military aid for as long as necessary" [7]). However, already in 2023 anecdotal evidence indicated challenges deriving from domestic politics for lasting support: in the US, factions among the Republican party positioned against arms deliveries to Ukraine; in Germany, a new leftist party formed in 2024, with the explicit aim to defund Ukraine military aid [see 8]; and the electoral campaign of Donald Trump for US president in 2024 created relevant ambiguity on the US governments' future position regarding Ukraine aid. Since early 2025, this culminated in repeated shifts in the position of the US presidency on Ukraine support, likewise in some European countries (e.g., Czechia after its 2025 elections[1]). For example, President Trump at times publicly emphasized the costs of support regarding the (US) economy, military and civilian death on the battleground, and escalation potential over Ukrainian sovereignty or territorial integrity – "Make a deal or we are out"[2] – while at other times considering heightened support for Ukraine.[3] Anecdotally, these shifts also reflect in public opinion polls showing increasing divergence in citizens' support for strong resolve.[4] From this perspective, citizen decisions at past and upcoming elections in the US and Europe are fundamental for the extent of resolve Western countries can actually show and how credible reassurance of security guarantees of these countries for Ukraine actually are [9, 10].

However, we know little on how public opinion understands the fundamental trade-offs involved in supporting Ukraine. The current state of the literature almost exclusively relies on observational research designs. Observational studies indicate that in the first year of the war, support for Ukraine aid across 16 EU countries and the UK was seemingly high, and stable [11], but not in all countries [12]. Studies indicate that, with the Russian aggression, support for more ambitious national defense policy increased [13–15], including for nuclear deterrence [16], though underlying foreign policy postures might not have changed [17]. Likewise, support for ambitious EU defense policy integration seems heightened [15, 18], with heterogeneity over countries [14, 19]. A small literature also started investigating polarization [20, 21]. Moise & Wang [20] indicate, as we do, that attitudes towards Russia/NATO play a relevant role in shaping attitudes.[5] Besides scholarly work, opinion polling by various think tanks, e.g., the Chicago Council on Global Affairs and by Pew Research Center, shed light on public opinion. The former investigates US public opinion, reporting strong majority support from US citizens for Ukraine aid (e.g., [23]). However, in interpreting this result, potential overstatements due to social desirability bias have to be taken into account, given that earlier surveys indicated relevant sensitivity to costs and security considerations among US citizens

[1] See https://www.politico.eu/article/czech-republic-to-slash-military-aid-to-ukraine-says-likely-next-fm-filip-turek/.

[2] White House Meeting between US President Trump and Ukrainian President Zelenskyy on February 28, 2025, see https://www.theguardian.com/us-news/2025/feb/28/trump-zelenskyy-meeting-transcript.

[3] https://edition.cnn.com/2025/09/23/politics/trump-nato-countries-russian-aircraft.

[4] E.g., the 2023 autumn Eurobarometer indicates that more than 30 percent of Europeans (tend to) disagree with financing or supplying military equipment to Ukraine (https://europa.eu/eurobarometer/surveys/detail/3053), up by about 3 percentage points (10%) compared to summer of the same year.

[5] See also [22] for correlational evidence on the association of attitudes towards Ukraine support and vote intentions.

[24], which can not be adequately captured in non-experimental surveys. The latter conduct cross-country opinion polling, partly indicating split public opinion on Ukraine aid (e.g., [25]), but do not directly investigate the trade-offs involved, which may limit citizens' overall support for Ukraine aid.

Survey-experimental work on mass public preferences subsequent to the Russian aggression in Ukraine in 2022, which can better approximate real-world decision-making of citizens [26], by directly confronting respondents with these trade-offs, and thereby also mitigating social desirability bias [27],[6] is, to our knowledge, only starting to appear (at the time of writing). Published survey-experimental work, for example, investigates Ukraine-related immigration politics [22, 31], prominently emphasizing the relevance of geo-political considerations in this regard [32]. Other work is still in the working paper stage, e.g., investigating support for sanction compliance with German respondents [33].

1.2 Comparison to the Pre-Registration

The pre-registration for our study was registered with the Open Science Foundation (OSF) and is available at `https://osf.io/tvzsa`. We uploaded the pre-registration to OSF on July 18, 2023. Data collection ran from June 14, 2023, to August 28, 2023. Data was collected through the survey company YouGov on its own survey platform. We explicitly agreed with YouGov not to hand over any data before registration to ensure proper blindness during finalization of the pre-registration. We shared proof of these timelines with the editors. Our pre-registration specified, on the one hand, core arguments and hypotheses, and on the other hand, our study design.

Study design

Our experimental design and analyses were conducted in accordance with the pre-registration. The relevant sections of the article (Methods section) and of the pre-registration (sections 4-8) map onto each other as follows:

- we proposed and implemented a complex multidimensional quota sample from the US, UK, French, German, and Italian online-access panels of YouGov (pre-registration section 4.1 and Methods section Sampling strategy);
- we proposed and implemented a quota and weight scheme to approximate population representativity (pre-registration section 4.2 and Methods section Sampling strategy);
- we proposed and implemented a conjoint experiment (pre-registration section 4.3 and Methods section Set-up of the conjoint experiment) and vignette experiment (pre-registration section 4.4 and Methods section Set-up of the vignette experiment);
- we proposed and implemented estimation of AMCEs and MMs, checking for robustness of the design with ACPs and AFCPs, also checking underlying assumptions of conjoint experiments by assessing profile and task order effects (pre-registration sections 6 and 8 and Methods section Estimation strategy and robustness);
- the estimated effects we interpret substantively are within our range of statistical power (pre-registration section 7).

[6]For example, observational studies on Russian public opinion after the war in Ukraine [28, 29] are likely severely overstating support for the regime [30].

Arguments and hypotheses

Regarding the argument and hypothesis section of the pre-registration (sections 2 and 3), we A) carried out all main analyses as planned; B) did not carry out all secondary analyses proposed in the pre-registration for reasons of publication space and manuscript coherence; and C) specifically focused in the article on the relevance of pro-/anti-Western attitudes as a mediating variable.

In the following, we report on how we followed the pre-registration, discuss secondary data analyses proposed in the pre-registration that did not make it into the manuscript, and explain (the few) deviations from the pre-registration for full transparency. Thereby, we follow the recommendation of Banerjee *et al.* [34] and provide a summary on the pre-registration, while also writing an article that is to be judged independently of our pre-registration.

Mapping of Figure 1 to pre-registration

Main Figure 1 presents our core expectations for the conjoint experiment in a very condensed form. The list below provides details on how the expectations detailed in Main Figure 1 (rightmost panel) map onto the pre-registration:

- Human costs: [Support] decreases with higher costs; more for human loss, but not for Russian casualties
  - pre-registration p. 4, conjoint hypothesis 4 (main expectation)
- Costs of aid: [Support] decreases with higher costs; hawks prefer military, all others economic aid
  - pre-registration p. 4, conjoint hypothesis 2 (main expectation)
  - pre-registration p. 5, trade-off hypothesis 2 (hawk subgroup expectation)
- Strategic risks: [Support] decreases with higher risk
  - pre-registration p. 4, conjoint hypothesis 3 (main expectation)
- Political costs/Containment of aggression (resolve): [Support] increases with lower costs; on average principled support, but not with anti-Western or pacifist attitudes
  - pre-registration p. 4, conjoint hypothesis 1 (main expectation)
  - pre-registration p. 5, trade-off hypothesis 1 (expectation of principled support, pro-Western subgroup expectation[7], pacifist expectation)

Mapping of Figure 5 to pre-registration

Main Figure 5 presents our core expectations for the vignette experiment in a very condensed form. The list below provides details on how the expectations presented in Main Figure 5 (rightmost panel) map onto the pre-registration:

- On average, support for offensive aid
  - pre-registration p. 5, vignette hypothesis 3
- Decreasing with aid's offensive potential
  - pre-registration p. 5, vignette hypothesis 1

[7]What we named pro-Russian attitudes in the pre-registration is named anti-Western attitudes in the article.

- Large offensive potential related to higher Ukrainian winning chances, larger human and economic costs, and strategic risks
  - pre-registration p. 5, vignette hypothesis 2

Summary on all proposed hypotheses and outlined analyses as worded in the pre-registration

Conjoint, main effects

As presented in the pre-registration, bottom of p.4, "concerning main effects in the conjoint experiment, we hypothesize that

1. At the margin, citizens strongly prefer full sovereignty for Ukraine (both regarding territorial concessions and political autonomy).
2. At the margin, citizens are cost-averse, i.e., costs of aid lead to lower support.
3. At the margin, citizens are risk-averse, i.e., increased nuclear threat leads to lower support.
4. At the margin, citizens despise increased military and civilian casualties and destruction, which hence lower support. The penalty for casualties and destruction is strongest for civilian casualties, followed by death of Ukrainian soldiers, death of Russian soldiers, and destruction of physical infrastructure."

Results for these expectations are presented in Main Figure 2:

- Regarding 1), we find evidence consistent with this hypothesis: significantly lower choice probabilities with attribute levels that show higher territorial concessions, or lower political autonomy.
- Regarding 2), we find evidence consistent with this hypothesis: significantly lower choice probabilities at higher costs for economic aid or military aid.
- Regarding 3), we find evidence consistent with this hypothesis: significantly lower choice probabilities at higher nuclear strike risk.
- Regarding 4), we find partial support for this hypothesis. Indeed, we see lower choice probabilities with increased military and civilian casualties and destruction, even though substantively small and insignificantly estimated for Russian military casualties. Regarding effect size comparison, we take the AMCE of the highest level, multiply it by the number of casualties at the reference level (lowest), and divide by the number of casualties at this (highest) level. This yields for civilian casualties a decrease in choice probabilities per percent increase of casualties of $-0.084[-0.074; -0.093]$; for Ukrainian military deaths: $-0.094[-0.085; -0.104]$; for Russian soldiers: $-0.007[-0.016; 0.002]$. For infrastructure damage, this yields $-0.032[-0.041; -0.023]$. Hence, comparing the change in choice probabilities in relation to the percentage change in casualty/damage increase indicates a preference ordering of preventing Ukrainian military death $\sim$ Ukrainian civilian death $\succ$ Ukrainian infrastructure damage $\succ$ Russian military death. Against our expectations, Ukrainian lives are valued similarly highly, independent of whether they are civilians or soldiers, while respondents barely care about the lives of Russian soldiers.

Deviations of the manuscript to the pre-registration are minor, only concerning a simplified formulation of expectation 4 (see Main Figure 1).

Conjoint, trade-offs

As presented in the pre-registration, top of p.5, "concerning trade-offs, we hypothesize that

1. Citizens are willing to bear large monetary, human, and strategic costs for upholding the sovereignty of Ukraine (indication of principled support). An exception should be respondents with pacifist (for moral reasons) and pro-Russian attitudes (for principled reasons). We will investigate to what extent citizens show indications for conditional support, i.e., whether they withdraw support for upholding the sovereignty of Ukraine when human, strategic, or monetary costs increase (indication of conditional support).

2. Citizens prefer economic over military aid if they have the choice, but not necessarily if they exhibit hawkish attitudes.

3. Citizens penalize infrastructure damage less than human casualties, military casualties less than civilian casualties, and Russian casualties less than Ukrainian casualties."

Results are presented in Main Figures 2, 4, and Supplementary Figure 7.

- Regarding 1), against expectations, we do not find strong evidence for principled support in the overall sample. As discussed in manuscript section *Western publics support Ukrainian territorial integrity and sovereignty, but take human costs and strategic risks into account*, "we observe that preferences are by no means unconditional (against our expectation in H4a). In our sample, average respondents place particular emphasis on the human costs of war and the potential for nuclear escalation, at levels comparable to those for extensive containment. This indicates overall a conditional calculus, limiting considerably the extent of resolve with larger human costs and strategic risks, though not economic costs." This finding is supported by our assessment of ACPs (see Supplementary Figure 15), which are particularly suited to compare attributes of varying numbers of levels. We proposed, however, that support for territorial integrity and political sovereignty would be lower among A) pacifist respondents and B) respondents with pro-Russian attitudes (which we term anti-Western in the article).

  For A), we find tentative support. Our emphasis on moral reasons pointed to the relevance of human death and destruction as a constraint on supporting Ukraine for pacifist respondents. As presented in Supplementary Figure 7, pacifist respondents show substantively stronger reactions to Ukrainian military and Ukrainian civilian casualties. While they do not show a decrease in support for Ukrainian political sovereignty (i.e., a similarly high change in choice probabilities for no territorial concessions and high political autonomy compared to non-pacifist respondents), the *relative* importance of upholding Ukrainian sovereignty is lower. This is confirmed by assessing ACPs (see Supplementary Figure 17).

  For B), we find strong evidence in line with our argument. Respondents in the top quartile of pro-Russian/anti-Western attitudes do not respond to varying levels of territorial concessions and are more likely to choose a neutral status for Ukraine than full sovereignty. This pattern of preferences indicates (principled) opposition to upholding Ukraine's full sovereignty. As a converse of this picture, respondents in the top quartile of pro-Western attitudes care strongest about the political sovereignty of Ukraine, the relative importance of this attribute indicating principled support almost *at any cost* (compare Main Figure 4 and Supplementary Figure 16 and corresponding manuscript discussion).

  Last, we proposed investigating whether respondents withdraw support for upholding Ukraine's sovereignty when human, strategic, or monetary costs increase as an indication of conditional support. Given that respondents show, on average, a conditional rather than a principled calculus from the start, testing this expectation was unnecessary for our aim of discerning whether citizens are really principled overall (which we could not confirm, as outlined above).

However, we investigated the converse: to what extent citizens show indications of principled support, and we found that respondents with strong pro-Western attitudes did so (see above).

- Regarding 2), we find no evidence that citizens, on average, prefer economic over military aid. This is inferred from the similarly sized decreases in choice probability with increases in military or economic aid (see Main Figure 2), both presented on an identical scale. Regarding the subexpectation that hawks show a stronger preference for military aid, we find tentative evidence consistent with it. As shown in Supplementary Figure 7, hawkish respondents are unresponsive to increases in military aid, whereas other respondents exhibit decreasing choice probabilities with higher levels of military aid.
- Regarding 3), we find partial evidence in line with our expectation. Comparing the change in choice probabilities in relation to the percentage change in casualty/damage increase indicates a preference ordering of preventing Ukrainian military death $\sim$ Ukrainian civilian death $\succ$ Ukrainian infrastructure damage $\succ$ Russian military death. In line with our expectations, increasing infrastructure damage is penalized less than (Ukrainian) human casualties. Also, Russian casualties are penalized relevant less than Ukrainian casualties. However, against our expectations, (Ukrainian) military casualties are penalized similarly strongly compared to civilian casualties.

Deviations of manuscript to pre-registration were as follows: regarding the last sentence of pre-registration expectation 1), given respondents show, on average, a conditional and not a principled calculus from the start, we did not investigate which citizens show a conditional, but rather which show a principled calculus (which we found for respondents with strong pro-Western attitudes). Also, we present pre-registration expectation 3) in a simplified form.

Vignette experiment

As presented in the pre-registration, middle of p.5, "Regarding the vignette experiment, we hypothesize that

1. Citizens, on average, are most supportive for the transfer of defensive weapons, where support decreases with offensive potential, and are unsupportive of own troop engagement and all-out sanctions.
2. Citizens relate stronger offensive weapons, all-out sanctions and own troop engagement with, on the one hand, better chances to win the war for Ukraine, but also longer fighting, more destruction, more refugees, a stronger risk of escalation, and economic disadvantages for the own country.
3. Citizens are, on average, in favor of offensive weapons, although they see the above-mentioned negative side-effects for Ukraine (suffering, refugees), also for their own country (risk of escalation, economic disadvantages, refugees)."

Results are presented in Main Figures 6 and 7.

- Regarding expectation 1), we find partial support. As shown in Figure 6, support increases significantly with more offensive potential (air defense, combat tanks, and fighter jets) than with more defensive potential (air defense only), contrary to our expectations. At the same time, one's own country's troop deployment sees, as expected, the lowest support. Again, against our expectations, all-out sanctions receive the highest support, both absolutely and relatively, compared to defensive/offensive weapons.

- Regarding 2), we find partial support. As presented in Main Figure 7, citizens indeed relate stronger offensive weapons, all-out sanctions, and own troop engagement with, on the one hand, better chances to win the war for Ukraine, a stronger risk of escalation, and economic disadvantages for the own country – but not longer fighting, more destruction, or more refugees.
- Regarding 3), we find tentative support: Respondents are, on average, in tentative support of offensive weapons (around 4.2 on the 7-point scale for the delivery of air defense, combat tanks, and fighter jets). However, against our expectations, they do not perceive such delivery as going hand in hand with more suffering in Ukraine or more refugees from Ukraine, and do not see increased economic disadvantages. They perceive a higher risk of escalation, however.

Deviations of manuscript to pre-registration were minor; we presented pre-registration expectations 1), 2), and 3) in a simplified form (see Figure 5).

Heterogeneity

Regarding effect heterogeneity, we proposed in the pre-registration (see lower section of p. 6) to investigate

1. "to what extent citizen reactions are structured by national contexts, i.e., whether we see different reactions in different country samples – prime candidates to explain these differences are, amongst others, country level state of the debates, country affectedness of economic repercussions, country level military expenditures and strategic cultures, the extent of polarization, or country level institutions."
2. "We will as well investigate to what extent certain subpopulations show differing attitudes, in particular those defined by attitudes towards foreign policy and war and peace, and by political and socio-demographics (prime candidates for the former are income; for the latter political orientations and party affiliations)."
3. "Last, we will investigate preferences by issue importance and perceptions of fear for the war in Ukraine, as detailed below (this might feed into a separate research manuscript given the extensive subgroup analyses involved)."

Results are presented in Main Figure 3 and Supplementary Figures 11 and 12.

- Regarding 1), we present evidence of heterogeneity and discuss it in the article section Cross-country comparison.
- Regarding 2), we chose a specific focus on heterogeneity by anti-/pro-Western attitudes of respondents, given, as we describe in the manuscript with more detail, "extensive exploratory analyses [...] indicate that pro-/anti-Western alignment is the major divide between citizens' positions regarding Ukraine policies" (see article section Subgroup analyses reveal heterogeneity). We still present heterogeneity by attitudes towards war and peace and foreign policy values in Supplementary Information section 3.3. There, we find that both attitudes towards war and peace (operationalized by two dimensions, and depicting the bottom and top quartiles of respondents on these dimensions; see Methods section Non-experimental variables and measures for details) and foreign policy values (operationalized by three dimensions, and depicting the bottom and top quartiles of respondents on these dimensions) meaningfully structure respondents preferences regarding Ukraine support strategies and their resolve. We find that across almost all attributes, a similar fundamental structure of preferences emerges among the bottom and top quartiles, as revealed for the overall sample with Main Figure 2. Exceptions are no statistically significant evidence for increases in choice probabilities for

military aid among respondents with high expression of isolationist values; and, likewise, none for aid increases among respondents with high expression of cooperative international values. Of course, the relative weight of attributes differs substantively and significantly between respondents with high/low expression of specific values/attitudes. In this manuscript, we refrain from interpreting these differences beyond what we have already discussed above regarding concrete hypotheses (e.g., for hawks or pacifists). In sum, the extent of heterogeneity by attitudes towards war and peace or foreign policy values is, however, dampened compared to the major divide in citizens' preferences: the extent to which they are rooted in the liberal world order.

- Regarding 2), heterogeneity by political attitudes and socio-demographics, and regarding 3), by issue salience and fear, we did not analyze and do not present heterogeneity in this manuscript. Concerning the former, this goes beyond the scope of the current manuscript, given the many possible ways by which such subgroup analyses could be conducted, as we did not specify concrete expectations or operationalizations – we propose future research investigates this by way of newly developed methods for subgroup analysis in conjoint experiments, allowing to explore this inductively [e.g., 35]. Note that we provide the respective data for these analyses with our replication data set. For the effects of the isolated moderator partisanship on Ukraine support strategies in the US subsample, see Rudolph [36]. Concerning the latter, issue salience and fear, this was already proposed for separate research in the pre-registration.

In summary, deviations of the article from the pre-registration were as follows: we chose a specific focus on heterogeneity by anti-/pro-Western attitudes of respondents for the article, while not analyzing heterogeneity by political attitudes and socio-demographics.

2 Supplementary Methods

2.1 Design of the Conjoint Experiment

Supplementary Table 1: The English-language wording of the nine conjoint attributes (nested in four dimensions) and the depicted attribute levels in the choice table

Dimension	Attributes	Level
Military casualties	Number of Ukrainian soldiers killed?	• 12,500<br>• 25,000<br>• 50,000
	Number of Russian soldiers killed?	• 25,000<br>• 50,000<br>• 100,000
Civilian death and destruction	Number of Ukrainian civilians killed?	• 4,000<br>• 8,000<br>• 16,000
	Value of destroyed infrastructure in Ukraine?	• $ [€,£] 50B<br>• $ [€,£] 100B<br>• $ [€,£] 200B
Aid	[Country] contribution to military aid to Ukraine?	• $ [€,£] [amount]B (ca. 0.1% of [country] economic output)<br>• $ [€,£] [amount]B (ca. 0.2% of [country] economic output)<br>• $ [€,£] [amount]B (ca. 0.3% of [country] economic output)
	[Country] contribution to economic aid to Ukraine?	• $ [€,£] [amount]B (ca. 0.1% of [country] economic output)<br>• $ [€,£] [amount]B (ca. 0.2% of [country] economic output)<br>• $ [€,£] [amount]B (ca. 0.3% of [country] economic output)
Nuclear risk	Risk of a Russian nuclear strike on Ukraine?	• Not present (0%)<br>• Low (5%)<br>• Moderate (10%)

Sovereignty	Territorial cessions of Ukraine to Russia?	• No cession of territories<br>• Cession of Crimea to Russia (ca. 4% of Ukrainian land)<br>• Cession of Crimea and separatist areas of 2014 to Russia (ca. 8% of Ukrainian land)<br>• Cession of Crimea and currently Russian-occupied territories to Russia (ca. 16% of Ukrainian land)
	How much political self-determination of Ukraine?	• Full self-determination (possibility of joining EU/NATO)<br>• Limited self-determination (no possibility of joining EU/NATO)<br>• Low self-determination (Russian influence on Ukrainian government)

Supplementary Table 2: Actual support levels for Ukraine between February 2022 and April 2023, based on Trebesch *et al.* [1].

Country	Total aid (sum of bilateral aid and share in EU assistance)	
	in billion €	in % GDP
France	7.81	0.32
Germany	14.68	0.41
Italy	5.50	0.31
United Kingdom	9.83	0.38
United States	71.28	0.36
average		0.36

Supplementary Table 3: Calculation of realistic levels of support in economic and military aid for the conjoint experiment. The table presents country figures for actual GDP∗0.18%, i.e., half the country aid average (second column), which are compared with half the total actual aid (see Suppl. Table 2) by country (third column). The last three columns present the actual levels chosen for the experiment, rounded for easier presentation to respondents.

Country	2022 GDP	Medium support as 0,18% x GDP	Actual total aid / 2	Medium rounded	**Low level: ca. 0.1%**	**Medium level: ca. 0.2%**	**High level: ca. 0.3%**
France	2,440.22	4.39	3.91	4	**2**	**4**	**6**
Germany	3,568.43	6.42	7.34	7	**3,5**	**7**	**10,5**
Italy	1,752.21	3.15	2.75	3	**1.5**	**3**	**4.5**
United Kingdom	2,560.35	4.61	4.92	5	**2.5**	**5**	**7.5**
United States	19,438.75	34.99	35.64	40	**20**	**40**	**60**

YouGov

We are now asking you for your first decision on the following two strategies. **The expected effects from today until the end of the war are given. Numerical values are given as forecasts over the next 12 months.**

	**Strategy A**	**Strategy B**
Territorial cessions of Ukraine to Russia?	Cession of Crimea and currently Russian-occupied territories to Russia (ca. 16% of Ukrainian land)	No cession of territories
How much political self-determination of Ukraine?	Low self-determination (Russian influence on Ukrainian government)	Low self-determination (Russian influence on Ukrainian government)
Number of Ukrainian civilians killed?	8,000	8,000
Value of destroyed infrastructure in Ukraine?	$100B	$100B
Risk of a Russian nuclear strike on Ukraine?	None (0%)	Moderate (10%)
U.S. contribution to economic aid to Ukraine?	$60B (ca. 0.3% of U.S. economic output)	$60B (ca. 0.3% of U.S. economic output)
U.S. contribution to military aid to Ukraine?	$20B (ca. 0.1% of U.S. economic output)	$40B (ca. 0.2% of U.S. economic output)
Number of Ukrainian soldiers killed?	12,500	25,000
Number of Russian soldiers killed?	100,000	25,000

If you had to choose between one of the two strategies, which one would you personally prefer?

Strategy A   Strategy B

Now consider both strategies individually.
On a scale of 1 to 7, where 1 means you would definitely support the strategy and 7 means you would definitely reject the strategy: **How would you rate the two strategies?**

	Strategy A	Strategy B
1 – Definitely support	○	○
2	○	○
3	○	○
4	○	○
5	○	○
6	○	○
7 – Definitely reject	○	○

>

Supplementary Figure 1: Look of the survey implementation of the conjoint experiment, the assessment of experimentally-varied Ukraine-support strategies. Screenshot of the conjoint experiment as shown on a desktop computer (example from the US survey). The rating section was only shown in the first two tasks.

2.2 Design of the Vignette Experiment

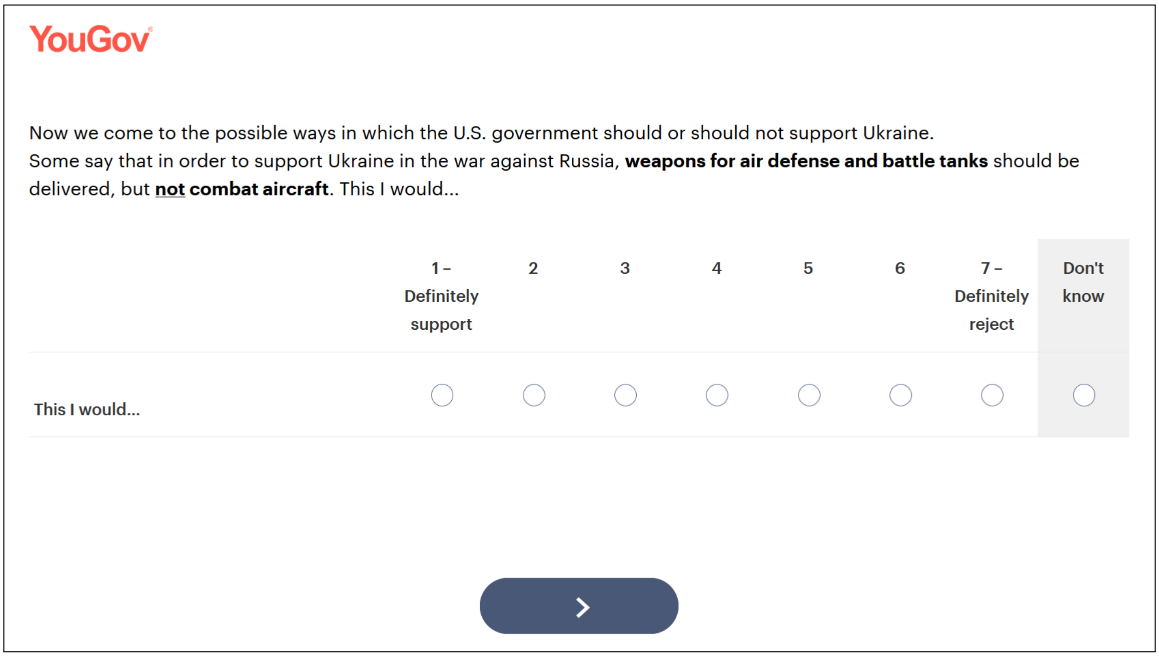

Supplementary Figure 2: Look of the survey implementation of the first part of the vignette experiment, agreement with government provision of aid types. Screenshot of the vignette experiment as shown on a desktop computer – first part (example from the US survey).

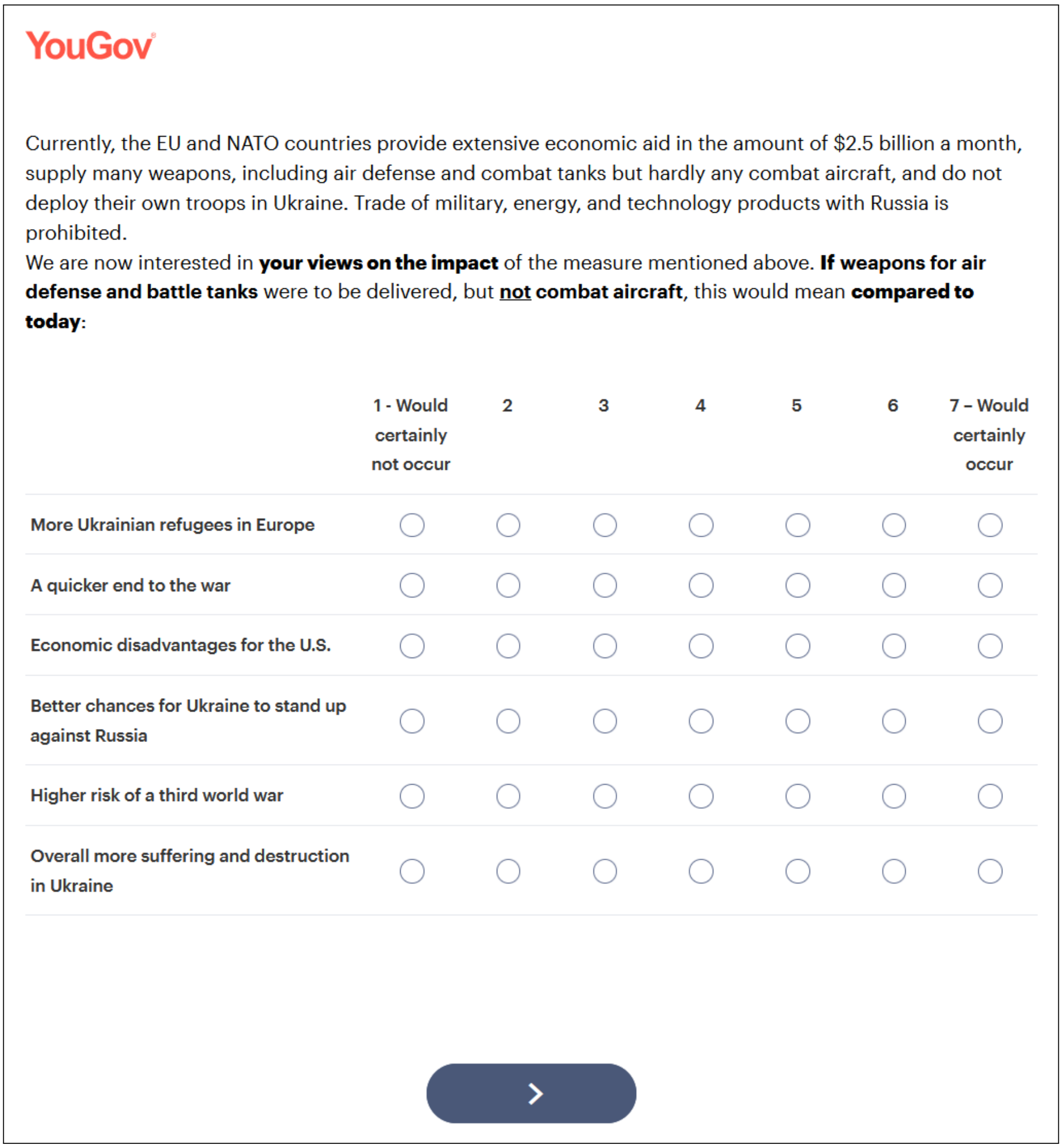

YouGov

Currently, the EU and NATO countries provide extensive economic aid in the amount of $2.5 billion a month, supply many weapons, including air defense and combat tanks but hardly any combat aircraft, and do not deploy their own troops in Ukraine. Trade of military, energy, and technology products with Russia is prohibited.
We are now interested in **your views on the impact** of the measure mentioned above. **If weapons for air defense and battle tanks** were to be delivered, but **not combat aircraft**, this would mean **compared to today**:

	1 - Would certainly not occur	2	3	4	5	6	7 – Would certainly occur
More Ukrainian refugees in Europe	○	○	○	○	○	○	○
A quicker end to the war	○	○	○	○	○	○	○
Economic disadvantages for the U.S.	○	○	○	○	○	○	○
Better chances for Ukraine to stand up against Russia	○	○	○	○	○	○	○
Higher risk of a third world war	○	○	○	○	○	○	○
Overall more suffering and destruction in Ukraine	○	○	○	○	○	○	○

Supplementary Figure 3: Look of the survey implementation of the second part of the vignette experiment, the battery on perceptions of consequences of aid types. Screenshot of the vignette experiment (second part) as shown on a desktop computer (example from the US survey).

3 Supplementary Results

3.1 Additional Results for Conjoint Experiment

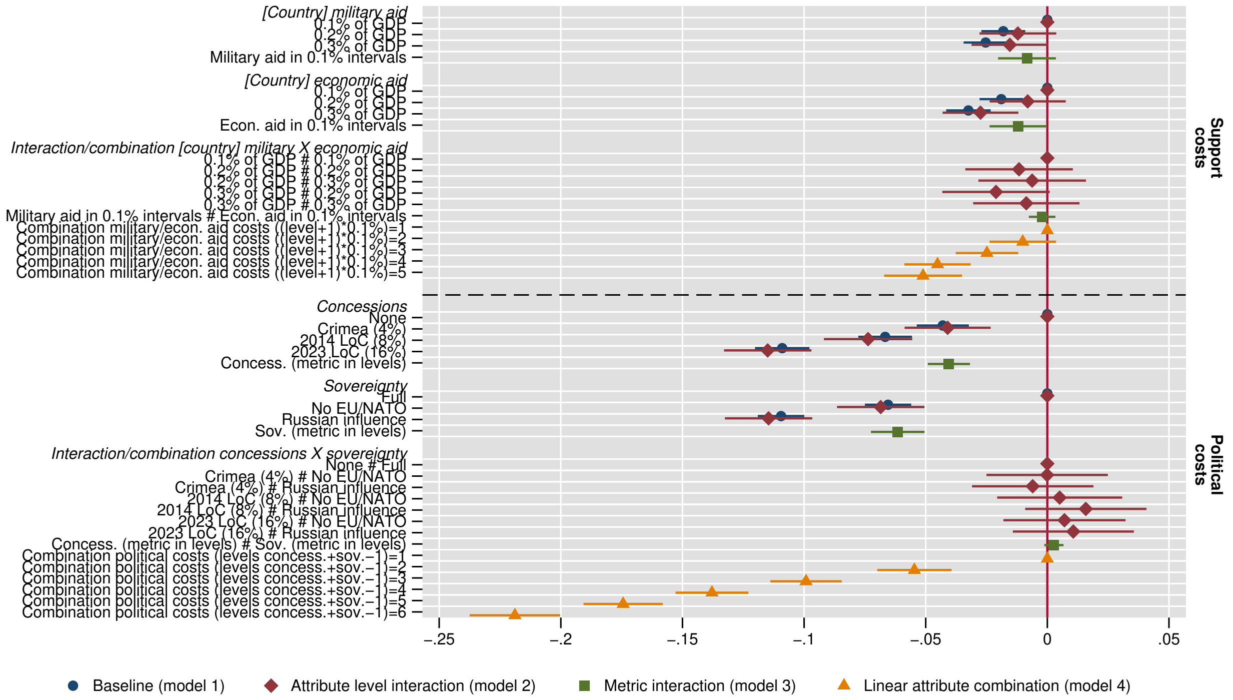

Supplementary Figure 4: Four models contrasting choice for tasks varying economic and political costs, averaging over all other attribute level expressions, indicate no evidence for statistically significant attribute level interaction effects within dimension. Model 1: Baseline AMCEs as presented in Main Figure 2 for the dimensions economic and political costs, i.e., linear regression of choice outcome on categorical indicators for economic and political attributes with theoretically most favorable level as baseline. Model 2: Addition of interaction term between categorical indicators within dimension. Model 3: Linear regression specified with metric attribute operationalization and its interaction within dimension. Model 4: Linear regression of choice outcome on combined indicator for joint distribution of attributes within dimension (sum of attribute levels within dimension - 1). 95% confidence intervals from respondent-clustered standard errors shown. $N = 80,088$ observations from $N = 10,011$ respondents. Degrees of freedom: $10,010$.

	(1)	(2)	(3)	(4)
	Anti-west att.	Anti-west att.	Anti-west att.	Anti-west att.
	b/p/ci95	b/p/ci95	b/p/ci95	b/p/ci95
Country indicator (ref.: US)				
Germany	-0.0384	0.0346	0.0333	-0.0557
	(0.4130)	(0.4751)	(0.6252)	(0.4230)
	[-0.1302,0.0535]	[-0.0603,0.1295]	[-0.1003,0.1669]	[-0.1921,0.0806]
Italy	0.2158	0.2699	0.2825	0.2694
	(0.0000)	(0.0000)	(0.0002)	(0.0005)
	[0.1154,0.3161]	[0.1614,0.3784]	[0.1316,0.4335]	[0.1185,0.4204]
UK	-0.1793	-0.1243	0.0076	-0.1113
	(0.0000)	(0.0029)	(0.8975)	(0.0570)
	[-0.2577,-0.1009]	[-0.2061,-0.0424]	[-0.1082,0.1234]	[-0.2260,0.0033]
France	0.0794	0.1294	0.1539	0.0894
	(0.0711)	(0.0078)	(0.0185)	(0.2050)
	[-0.0068,0.1657]	[0.0340,0.2248]	[0.0258,0.2819]	[-0.0489,0.2277]
Age group indicator (ref.: 18-30)				
31-45	0.0253	-0.0065	0.0137	-0.0583
	(0.5895)	(0.8944)	(0.8345)	(0.3853)
	[-0.0666,0.1172]	[-0.1020,0.0891]	[-0.1147,0.1420]	[-0.1899,0.0733]
46-60	-0.1018	-0.1316	-0.2497	-0.0490
	(0.0290)	(0.0070)	(0.0001)	(0.4668)
	[-0.1933,-0.0104]	[-0.2272,-0.0360]	[-0.3769,-0.1225]	[-0.1812,0.0831]
61-100	-0.3402	-0.3718	-0.3904	-0.3818
	(0.0000)	(0.0000)	(0.0000)	(0.0000)
	[-0.4479,-0.2325]	[-0.4869,-0.2568]	[-0.5456,-0.2353]	[-0.5421,-0.2214]
Gender (ref.: female)				
Male	-0.0006	-0.0087	0.0480	-0.0193
	(0.9821)	(0.7743)	(0.2425)	(0.6560)
	[-0.0567,0.0555]	[-0.0679,0.0506]	[-0.0325,0.1284]	[-0.1040,0.0655]
Education levle (ref.: low)				
Medium education	-0.0555	-0.0323	-0.0092	-0.0439
	(0.1195)	(0.4064)	(0.8574)	(0.4210)
	[-0.1255,0.0144]	[-0.1085,0.0439]	[-0.1093,0.0910]	[-0.1510,0.0631]
High education	-0.1874	-0.1465	-0.0432	-0.1368
	(0.0000)	(0.0007)	(0.4649)	(0.0197)
	[-0.2634,-0.1114]	[-0.2315,-0.0614]	[-0.1593,0.0728]	[-0.2518,-0.0218]
Employment status (ref.: working)				
Unemployed	0.0267	0.0352	0.0671	0.0212
	(0.6513)	(0.5658)	(0.3946)	(0.8084)
	[-0.0892,0.1426]	[-0.0849,0.1553]	[-0.0874,0.2217]	[-0.1498,0.1921]
Retired	-0.0600	-0.0431	-0.1028	0.0081
	(0.2243)	(0.4032)	(0.1320)	(0.9099)
	[-0.1567,0.0368]	[-0.1442,0.0580]	[-0.2365,0.0310]	[-0.1327,0.1490]
Other	-0.0476	-0.0646	-0.0475	-0.0144
	(0.2619)	(0.1614)	(0.4541)	(0.8202)
	[-0.1309,0.0356]	[-0.1551,0.0258]	[-0.1717,0.0768]	[-0.1384,0.1096]
Personal state of econ. (1 good - 5 bad)	0.0533	0.0530	0.0226	0.0892
	(0.0014)	(0.0024)	(0.3400)	(0.0002)
	[0.0205,0.0862]	[0.0187,0.0873]	[-0.0239,0.0692]	[0.0417,0.1366]
Country state of econ. (1 good - 5 bad)	0.2059	0.2005	0.1276	0.1807
	(0.0000)	(0.0000)	(0.0000)	(0.0000)
	[0.1715,0.2404]	[0.1649,0.2362]	[0.0780,0.1772]	[0.1311,0.2303]
Religion (ref.: none)				
Protestant	-0.1605	-0.1792	-0.1634	-0.1675
	(0.0001)	(0.0000)	(0.0034)	(0.0035)
	[-0.2384,-0.0826]	[-0.2590,-0.0994]	[-0.2726,-0.0542]	[-0.2798,-0.0553]
Roman Catholic	-0.0356	-0.0571	-0.0252	-0.1020
	(0.3344)	(0.1432)	(0.6172)	(0.0676)
	[-0.1079,0.0367]	[-0.1335,0.0193]	[-0.1239,0.0736]	[-0.2114,0.0074]
Orthodox Christian	0.5067	0.6312	0.5919	0.6621
	(0.0027)	(0.0002)	(0.0046)	(0.0041)
	[0.1760,0.8373]	[0.3014,0.9611]	[0.1823,1.0015]	[0.2099,1.1143]
Muslim	0.4505	0.4295	0.4888	0.3210
	(0.0000)	(0.0000)	(0.0005)	(0.0095)
	[0.2707,0.6304]	[0.2360,0.6230]	[0.2126,0.7650]	[0.0784,0.5636]
Jewish	-0.1021	-0.1900	0.1141	-0.3471
	(0.5053)	(0.1790)	(0.5767)	(0.0562)
	[-0.4024,0.1983]	[-0.4672,0.0871]	[-0.2867,0.5150]	[-0.7034,0.0092]
Other	0.0850	0.0873	0.1503	0.0411
	(0.2387)	(0.2571)	(0.1467)	(0.6963)
	[-0.0564,0.2264]	[-0.0637,0.2384]	[-0.0527,0.3532]	[-0.1652,0.2474]
Left-right positioning (ref.: left)				
Middle		-0.0165	-0.1239	-0.0363
		(0.6524)	(0.0145)	(0.4811)
		[-0.0882,0.0552]	[-0.2233,-0.0245]	[-0.1373,0.0647]
Right		0.1977	-0.0210	0.1601
		(0.0000)	(0.7355)	(0.0066)
		[0.1129,0.2825]	[-0.1429,0.1009]	[0.0445,0.2756]
Pol. participation (ref.: non-voter)				
Voter		-0.2143	-0.1986	-0.1999
		(0.0000)	(0.0003)	(0.0005)
		[-0.2956,-0.1330]	[-0.3066,-0.0905]	[-0.3124,-0.0875]
Political interest (1 low - 4 high)		0.0088	0.0351	0.0251
		(0.6166)	(0.1342)	(0.3125)
		[-0.0258,0.0435]	[-0.0108,0.0809]	[-0.0236,0.0739]
Isolationist att.			0.3717	
			(0.0000)	
			[0.3258,0.4175]	
Militant interv. att.			-0.1673	
			(0.0000)	
			[-0.2141,-0.1204]	
Coop. int. att.			-0.0385	
			(0.0306)	
			[-0.0734,-0.0036]	
Att. war/peace 1				0.1045
				(0.0000)
				[0.0705,0.1385]
Att. war/peace 2				0.0983
				(0.0000)
				[0.0594,0.1372]
Constant	-0.6863	-0.5945	-0.2516	-0.6761
	(0.0000)	(0.0000)	(0.0597)	(0.0000)
	[-0.8488,-0.5238]	[-0.7882,-0.4008]	[-0.5135,0.0103]	[-0.9451,-0.4071]
Observations	8713	7899	3926	3973
Degrees of freedom	8691	7873	3897	3945
R-squared	0.075	0.086	0.196	0.093
Sample mean	-0.010	-0.040	-0.014	-0.066
Sample standard deviation	1.282	1.294	1.290	1.297

Supplementary Table 4: Estimates (b) from regression of anti-Western attitudes (PCA score) on socio-demographics (model 1), as well as political attitudes (model 2), as well as foreign policy values (PCA scores) (model 3) or pacifist attitudes (PCA scores) (model 4). Robust standard errors used, p-values (p) and 95% confidence interval in parentheses.

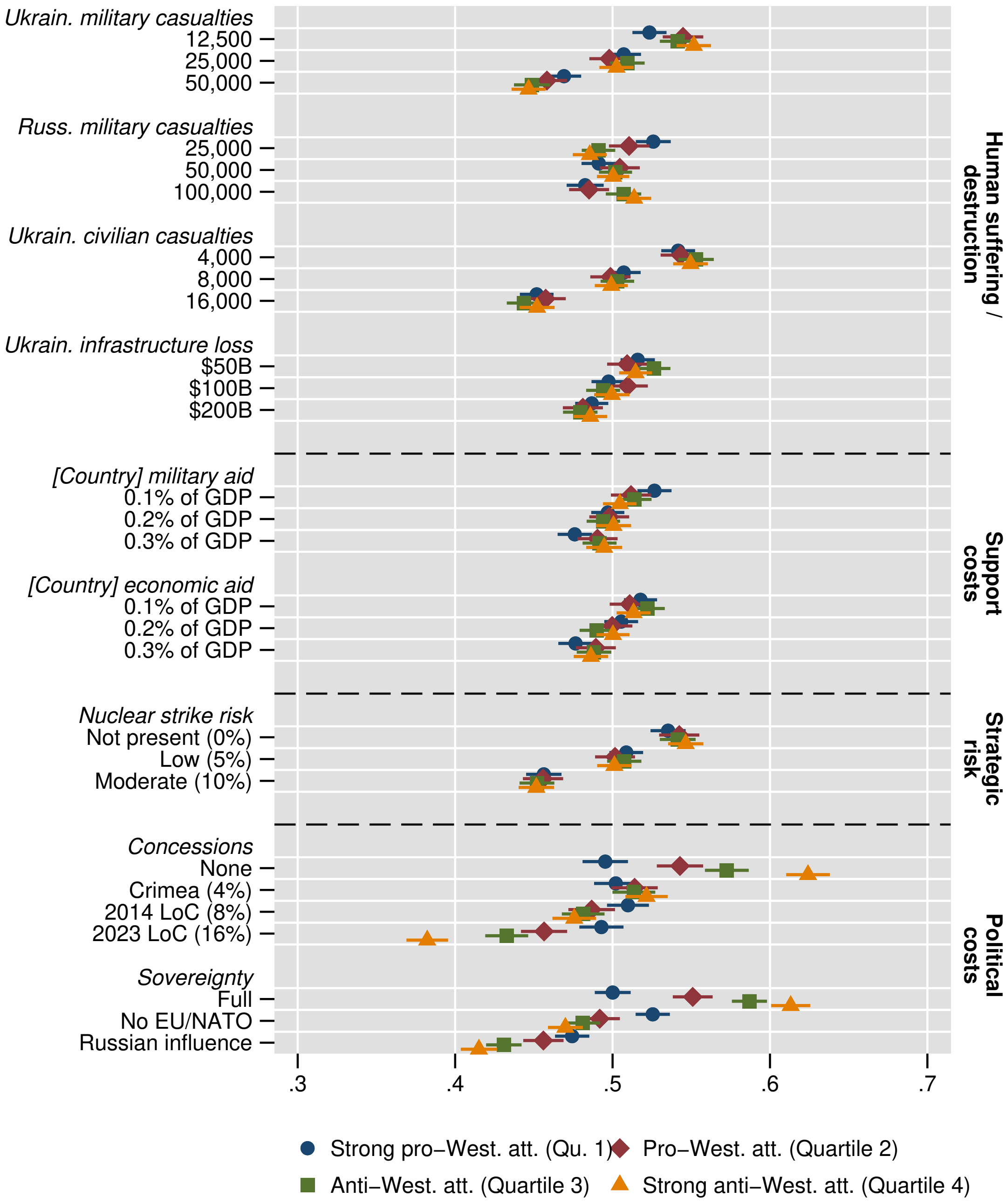

Supplementary Figure 5: Attribute favorabilities of intermediate quartiles of anti-/pro-Western attitudes are situated between extreme quartiles as presented in Figure 4. MMs for choice task by quartiles of anti-/pro-Western attitudes, including intermediate groups excluded from presentation in Main Figure 4. Blue circles and yellow triangles correspond to results presented in Main Figure 4. 95% confidence intervals from respondent-clustered standard errors shown. Estimates presented are predictions for support from linear regressions of choice on attribute-level indicators within attitude subgroups. See description of Main Figure 4 for details. Observations are $N_{\text{Quartile 1}} = 18,936$, $N_{\text{Quartile 2}} = 17,288$, $N_{\text{Quartile 3}} = 18,072$, $N_{\text{Quartile 4}} = 18,472$ from $N_{\text{Quartile 1}} = 2,367$, $N_{\text{Quartile 2}} = 2,161$, $N_{\text{Quartile 3}} = 2,259$, $N_{\text{Quartile 4}} = 2,309$ respondents, i.e., degrees of freedom are $df_{\text{Quartile 1}} = 2,366$, $df_{\text{Quartile 2}} = 2,160$, $df_{\text{Quartile 3}} = 2,258$, $df_{\text{Quartile 4}} = 2,308$.

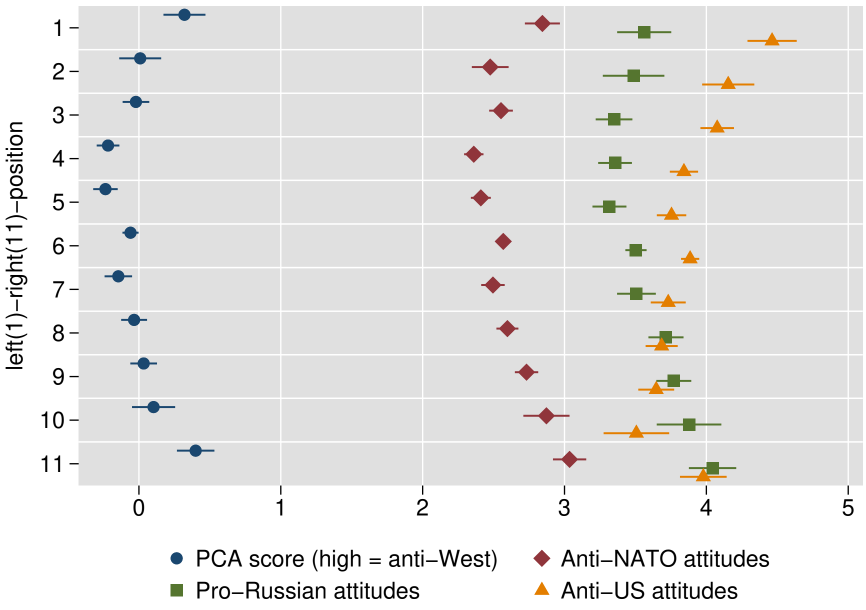

Supplementary Figure 6: The correlation of anti-Western attitudes (PCA scores) and of components of PCA index with left-right position of respondents indicates a u-formed relationship. Coefficients are predictions of left-right score based on a bivariate regression of attitudinal measure on categorical left-right positioning with 95% confidence intervals from robust standard errors. Left-right scales rescaled to common 11-point scale for all countries. $N_{\text{PCA score}} = N_{\text{Anti-Nato attitudes}} = 8,368$, $N_{\text{Pro-Russian attitudes}} = N_{\text{Anti-US attitudes}} = 9,034$ observations/respondents, i.e., $df_{\text{PCA score}} = df_{\text{Anti-Nato attitudes}} = 8,357$, $df_{\text{Pro-Russian attitudes}} = df_{\text{Anti-US attitudes}} = 9,023$ degrees of freedom.

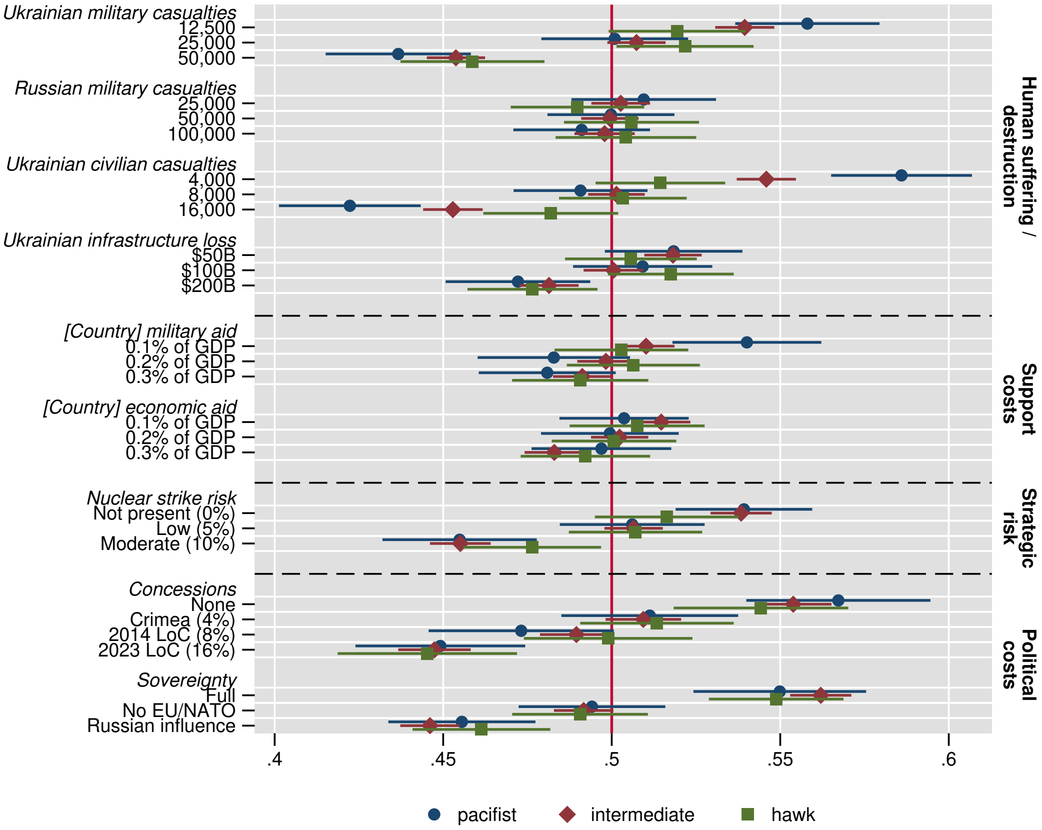

Supplementary Figure 7: Choice probabilities by pacifistic and hawkish respondents indicate heterogeneity in subgroup preferences for several attributes. MMs for choice task for pacifistic and hawkish respondents. The former are operationalized by the top quartile of the attitudes toward peace *and* bottom quartile of the attitudes war scale; the latter by the inverse. MMs for intermediate respondents are also shown. Attitudes towards war and peace are derived from the first two components of the item battery. Top (bottom) quartile has a particularly favorable (disfavorable) view on the relevance of peace (war). Scale inquired for a random subset of 50% of all respondents. 95% confidence intervals from cluster-robust standard errors shown. Estimates presented are predictions for support from linear regressions of choice on attribute-level indicators within attitude subgroups. $N_{\text{pacifist}} = 5,480$, $N_{\text{intermediate}} = 29,040$, $N_{\text{hawk}} = 5,616$ observations from $N_{\text{pacifist}} = 685$, $N_{\text{intermediate}} = 702$, $N_{\text{hawk}} = 3,630$ respondents, i.e., with $df_{\text{pacifist}} = 684$, $df_{\text{intermediate}} = 701$, $df_{\text{hawk}} = 3,629$ degrees of freedom.

3.2 Additional Results for Vignette Experiment

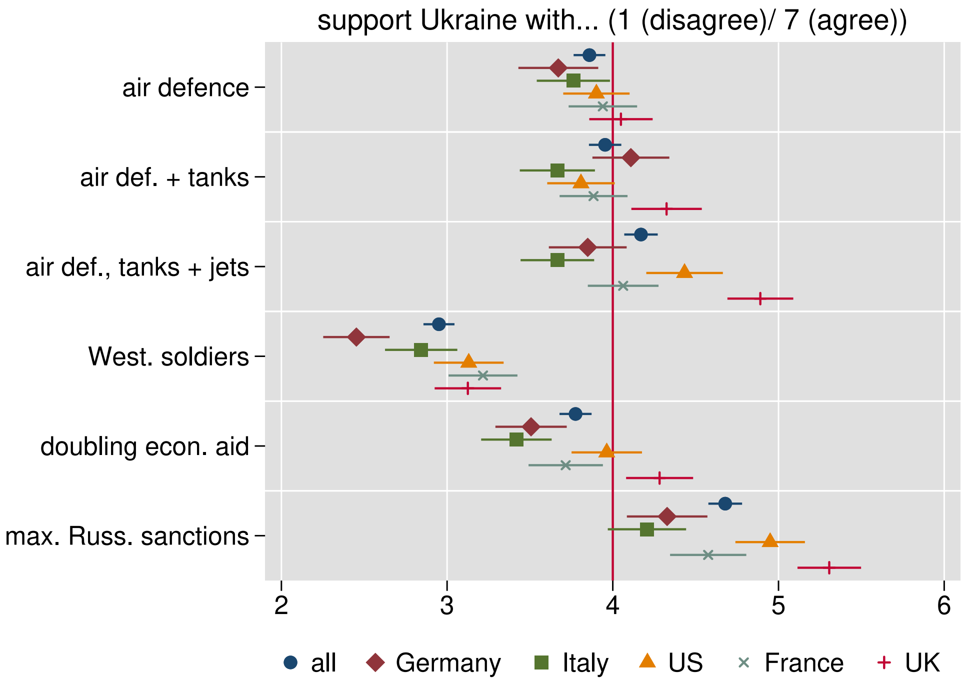

Supplementary Figure 8: Average agreement for different aid types by country subsample indicates country heterogeneity for several aid types. Marginal means by vignette conditions and country. 95% confidence intervals from respondent-clustered standard errors shown. Estimates presented are predictions from linear regressions of aid support on vignette conditions within country subgroups. Blue circles correspond to the full sample results presented in Main Figure 6. $N_{\text{all}} = 8,596$, $N_{\text{Germany}} = 1,804$, $N_{\text{Italy}} = 1,723$, $N_{\text{US}} = 1,732$, $N_{\text{France}} = 1,642$, $N_{\text{UK}} = 1,695$ respondents/observations, i.e., $df_{\text{all}} = 8,590$, $df_{\text{Germany}} = 1,798$, $df_{\text{Italy}} = 1,717$, $df_{\text{US}} = 1,726$, $df_{\text{France}} = 1,636$, $df_{\text{UK}} = 1,689$ degrees of freedom.

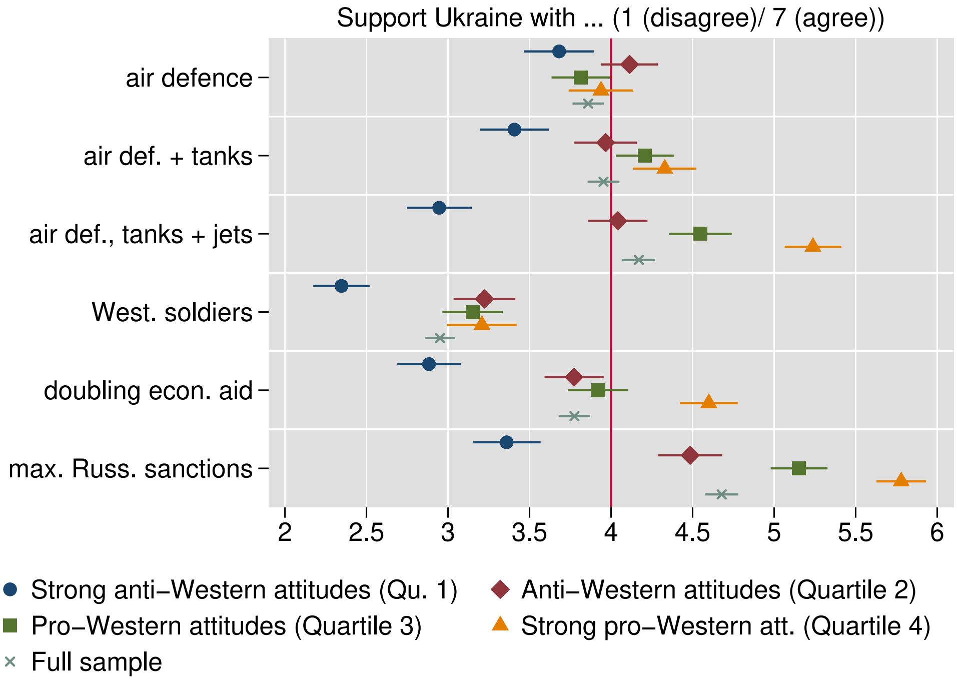

Supplementary Figure 9: Average agreement for different aid types by subgroups of respondents with differing extent of pro-/anti-Western attitudes indicates subgroup heterogeneity for several aid types. Marginal means by vignette conditions and subgroup. 95% confidence intervals from respondent-clustered standard errors shown. Estimates presented are predictions from linear regressions of aid support on vignette conditions within attitude subgroups. Grey crosses correspond to the full sample results presented in Main Figure 6. $N_{\text{Full sample}} = 8,596$, $N_{\text{Quartile 1}} = 2,170$, $N_{\text{Quartile 2}} = 1,876$, $N_{\text{Quartile 3}} = 1,994$, $N_{\text{Quartile 4}} = 2,149$ respondents/observations, i.e., $df_{\text{Full sample}} = 8,590$, $df_{\text{Quartile 1}} = 2,164$, $df_{\text{Quartile 2}} = 1,870$, $df_{\text{Quartile 3}} = 1,988$, $df_{\text{Quartile 4}} = 2,143$ degrees of freedom.

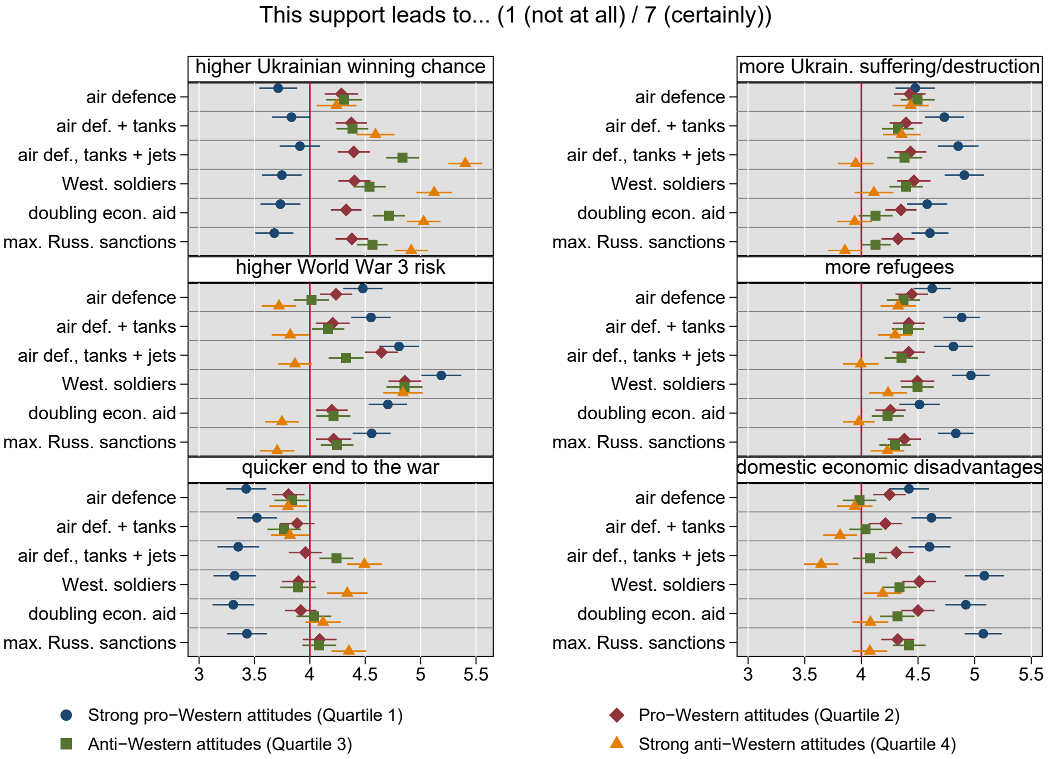

Supplementary Figure 10: Average perceived consequences of different aid types by subgroups of respondents with differing extent of pro-/anti-Western attitudes indicates subgroup heterogeneity for several aid types and perception measures. Marginal means for the perception of consequences (as indicated in panel header) for six different types of military and economic aid to Ukraine based on the split-sample aid vignette by subgroups of the first, second, third, and fourth quartiles of pro-/anti-Western attitudes. Error bars denote 95% confidence intervals from robust standard errors. Estimates presented are predictions for consequences based on separate linear regressions for each dependent variable on the vignette condition indicators within subgroups. Blue circles and yellow triangles correspond to results presented in Main Figure 8. Within each panel, $N_{\text{Quartile 1}} = 2,367$, $N_{\text{Quartile 2}} = 2,161$, $N_{\text{Quartile 3}} = 2,259$, $N_{\text{Quartile 4}} = 2,309$ respondents/observations, i.e., $df_{\text{Quartile 1}} = 2,361$, $df_{\text{Quartile 2}} = 2,155$, $df_{\text{Quartile 3}} = 2,253$, $df_{\text{Quartile 4}} = 2,303$ degrees of freedom.

3.3 Heterogeneity by Attitudes Towards War and Peace and Foreign Policy Values

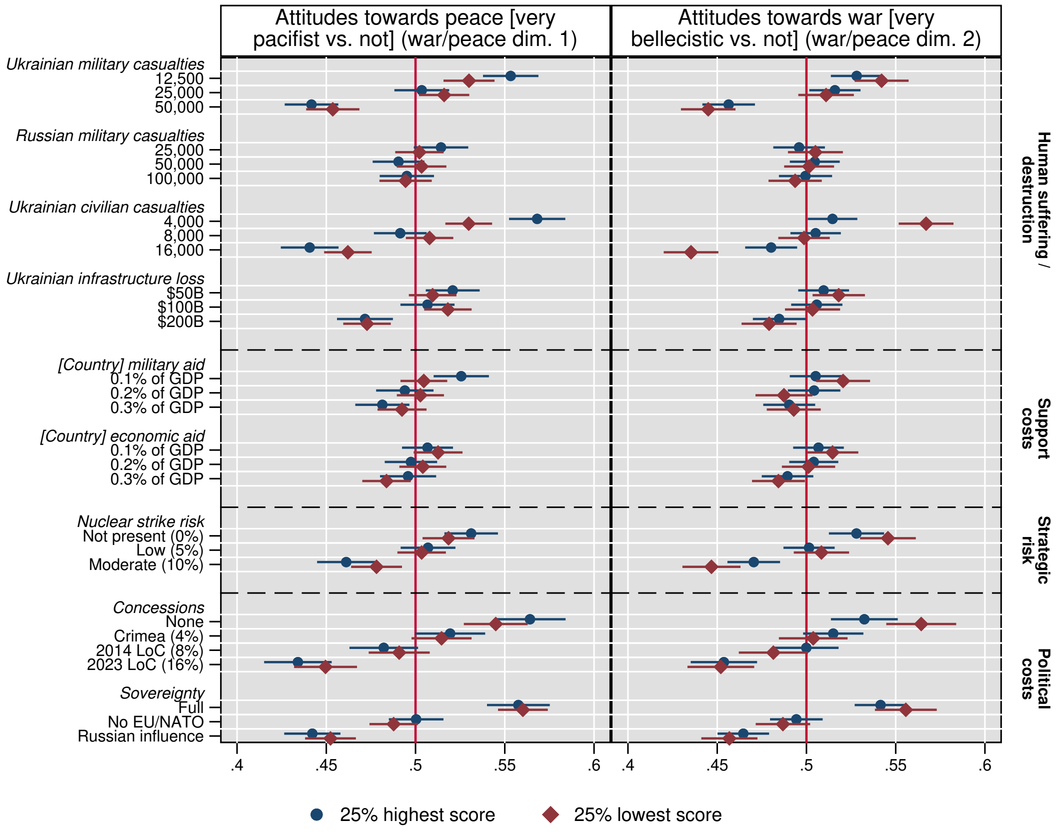

Supplementary Figure 11: Choice probabilities by extreme groups regarding attitudes towards peace/war indicate heterogeneity in subgroup preferences for several attributes. MMs for choice task by bottom/top quartile of first two components of the attitudes towards war and peace item battery (see panel header). Top (bottom) quartile has a particularly favorable (disfavorable) view on relevance of peace (war). MMs for intermediate respondents not shown. Scale inquired for a random subset of 50% of all respondents. 95% confidence intervals from respondent-clustered standard errors shown. Estimates presented are predictions for support from linear regressions of choice on attribute-level indicators within attitude subgroups. Left panel: $N_{25\% \text{ highest score}} = 10,040$, $N_{25\% \text{ lowest score}} = 11,664$ observations from $N_{25\% \text{ highest score}} = 1,255$, $N_{25\% \text{ lowest score}} = 1,458$ respondents, i.e., with $df_{25\% \text{ highest score}} = 1,254$, $df_{25\% \text{ lowest score}} = 1,457$ degrees of freedom. Right panel: $N_{25\% \text{ highest score}} = 10,632$, $N_{25\% \text{ lowest score}} = 10,040$ from $N_{25\% \text{ highest score}} = 1,329$, $N_{25\% \text{ lowest score}} = 1,255$ respondents, i.e., with $df_{25\% \text{ highest score}} = 1,328$, $df_{25\% \text{ lowest score}} = 1,254$ degrees of freedom.

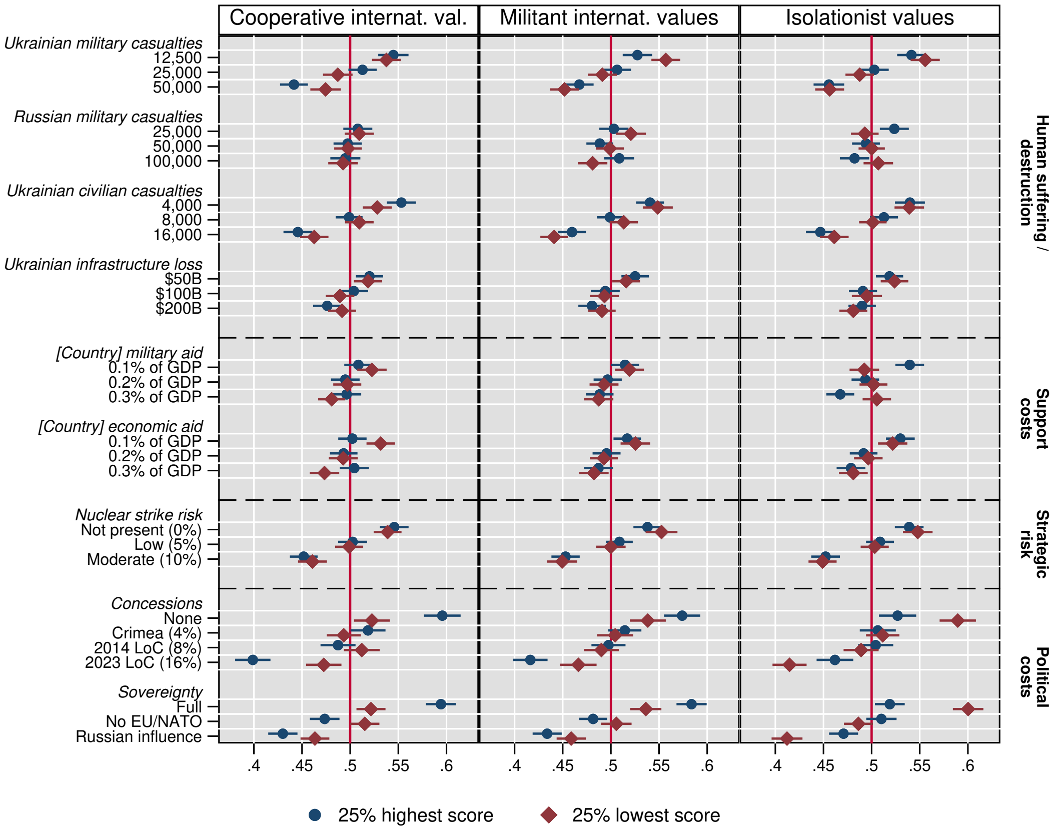

Supplementary Figure 12: Choice probabilities by extreme groups of foreign policy values (measured in three dimensions) indicate heterogeneity in subgroup preferences for several attributes. MMs for choice task by foreign policy values (see panel header for dimension). Top (bottom) quartile has a particularly high (low) expression of corresponding attitudes. MMs for intermediate respondents not shown. Scale inquired for a random subset of 50% of all respondents. 95% confidence intervals from respondent-clustered standard errors shown. Estimates presented are predictions for support from linear regressions of choice on attribute-level indicators within attitude subgroups. Left panel: $N_{25\%\text{ highest score}} = 9{,}992$, $N_{25\%\text{ lowest score}} = 9{,}992$ observations from $N_{25\%\text{ highest score}} = 1{,}249$, $N_{25\%\text{ lowest score}} = 1{,}249$ respondents, i.e., with $df_{25\%\text{ highest score}} = 1{,}248$, $df_{25\%\text{ lowest score}} = 1{,}248$ degrees of freedom. Mid panel: $N_{25\%\text{ highest score}} = 10{,}168$, $N_{25\%\text{ lowest score}} = 9{,}992$ observations from $N_{25\%\text{ highest score}} = 1{,}271$, $N_{25\%\text{ lowest score}} = 1{,}249$ respondents, i.e., with $df_{25\%\text{ highest score}} = 1{,}270$, $df_{25\%\text{ lowest score}} = 1{,}248$ degrees of freedom. Right panel: $N_{25\%\text{ highest score}} = 9{,}992$, $N_{25\%\text{ lowest score}} = 10{,}016$ observations from $N_{25\%\text{ highest score}} = 1{,}249$, $N_{25\%\text{ lowest score}} = 1{,}252$ respondents, i.e., with $df_{25\%\text{ highest score}} = 1{,}248$, $df_{25\%\text{ lowest score}} = 1{,}251$ degrees of freedom.

3.4 Robustness Checks

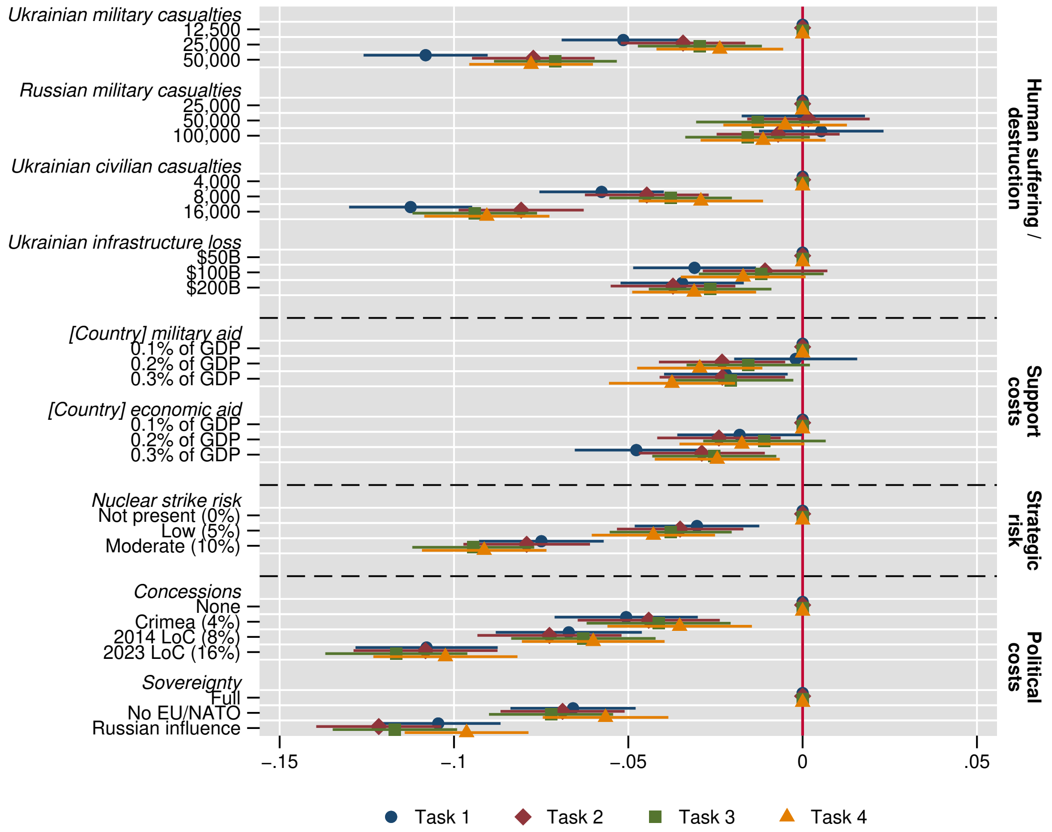

Supplementary Figure 13: Differentiated results by task number indicate little credible evidence of heterogeneity by task subsets. AMCEs for the conjoint choice task, estimated within subgroups of task order (task 1: presented first; to task 4: presented last). 95% confidence intervals from robust standard errors shown. $N_{\text{Task 1}} = N_{\text{Task 2}} = N_{\text{Task 3}} = N_{\text{Task 4}} = 20,022$ observations from $N_{\text{Task 1}} = N_{\text{Task 2}} = N_{\text{Task 3}} = N_{\text{Task 4}} = 10,011$ respondents, i.e., with $df_{\text{Task 1}} = df_{\text{Task 2}} = df_{\text{Task 3}} = df_{\text{Task 4}} = 10,010$ degrees of freedom.
AMCEs are highly comparable; we find no statistical evidence of differences by task order. Note that marginal means are likewise very comparable. With Bonferroni adjustment for multiple comparisons, none of the differences are statistically significant.

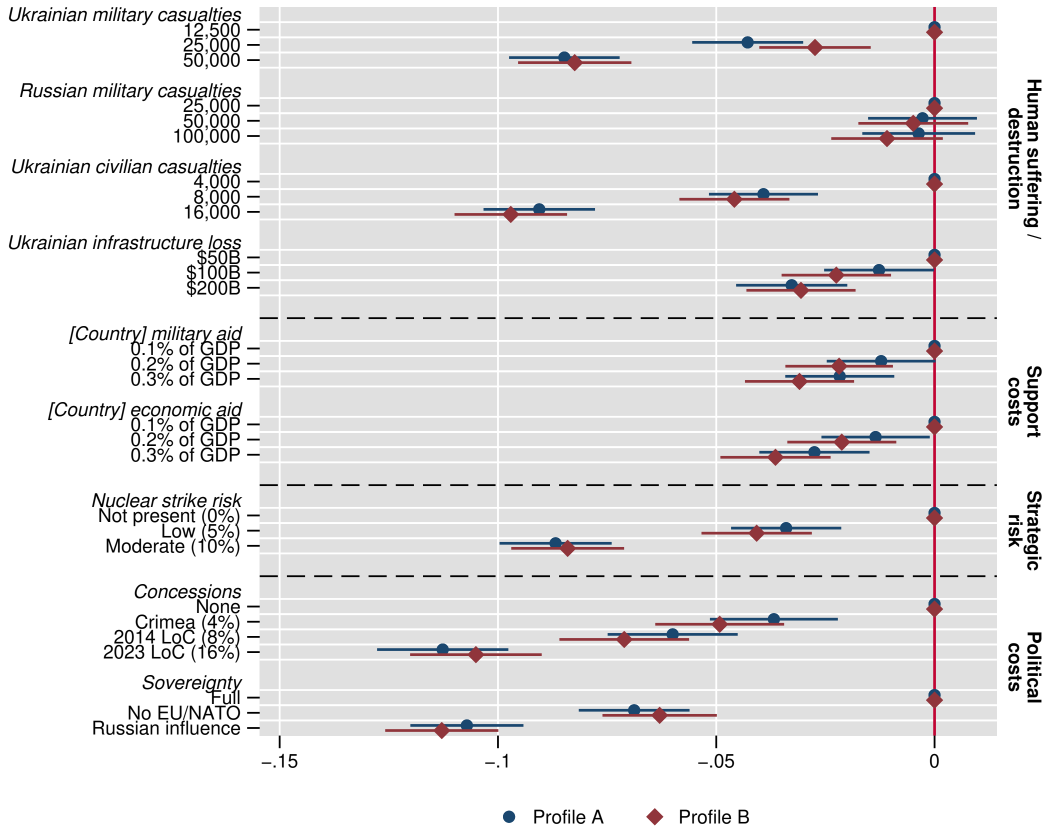

Supplementary Figure 14: Differentiated AMCEs by profile order indicate little credible evidence of heterogeneity by profile order subsets. AMCEs for the conjoint choice task, estimated within subgroups of profile order (profile A: presented on the left side; profile B: presented on the right side). 95% confidence intervals with respondent-clustered standard errors shown. $N_{\text{Profile A}} = N_{\text{Profile B}} = 40,044$ observations from $N_{\text{Profile A}} = N_{\text{Profile B}} = 10,011$ respondents, i.e., with $df_{\text{Profile A}} = df_{\text{Profile B}} = 10,010$ degrees of freedom.

AMCEs are highly comparable; we find no statistical evidence of differences by profile order. Note that marginal means indicate a difference in levels (but not in changes), with choice more likely on the left than on the right.

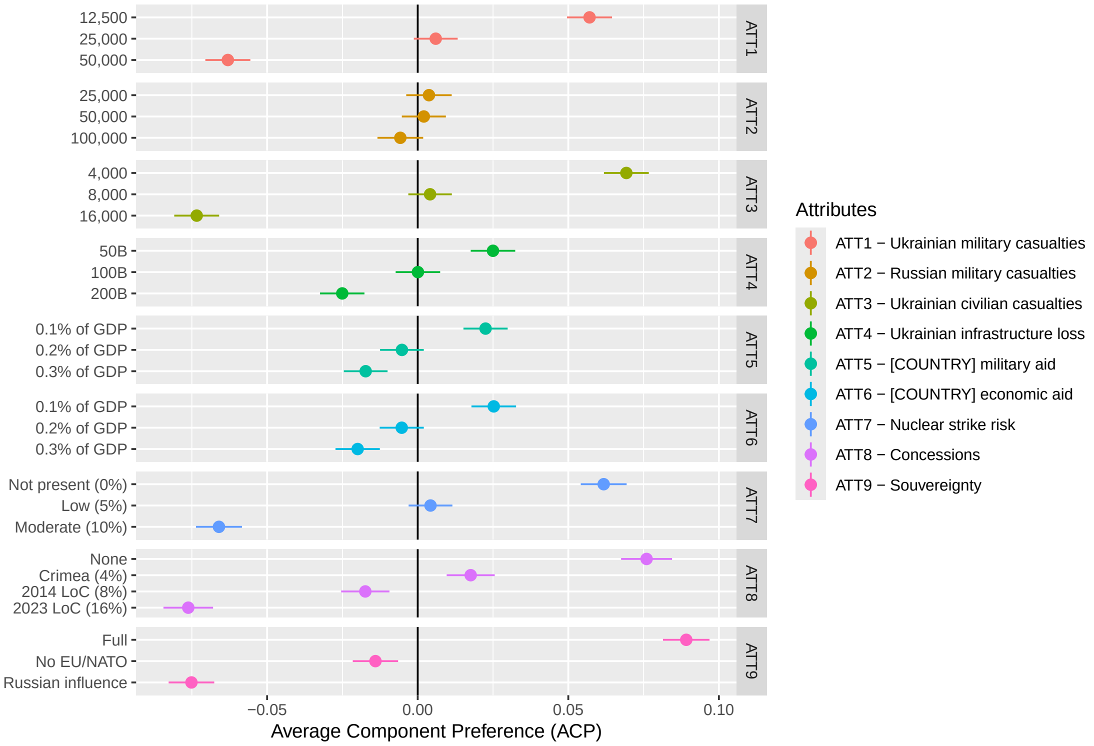

Supplementary Figure 15: Average Component Preferences (ACPs) for the conjoint choice task indicate a statistically significantly and substantively higher weight for attributes 1 (Ukrainian soldiers), 3 (Ukrainian civilians), and 7-9 (strategic risk and political cost attributes) compared to 5, 6 (support cost attributes), 4 (infrastructure damage), and 2 (Russian soldiers) in preference formation. 95% confidence intervals, based on robust standard errors, are shown. $N = 80,088$ observations from $10,011$ respondents ($df = 10,010$). Interpretation example for the ACP for a strategy with 16,000 Ukrainian civilian casualties of -0.073: When compared to a strategy with fewer civilian casualties, a strategy with 16,000 civilian deaths has a 42.7% (0.5-0.073) chance to be chosen. This can be contrasted to a strategy with 4,000 civilian casualties on the Ukrainian side, which has a 56.9% (0.5+0.069) chance to be selected, compared to strategies with more civilian victims. An ACP of 0 signals a situation of indifference (where the selection probability is 0.5), like it is almost the case for strategies with 8,000 Ukrainian civilian casualties.

A more holistic interpretation shows that a strategy with 16,000 Ukrainian civilian casualties has a roughly similar effect on respondents' choice probabilities as a strategy that causes Ukraine to lose about 16% of its territory or forces it to accept Russian influence on its government.

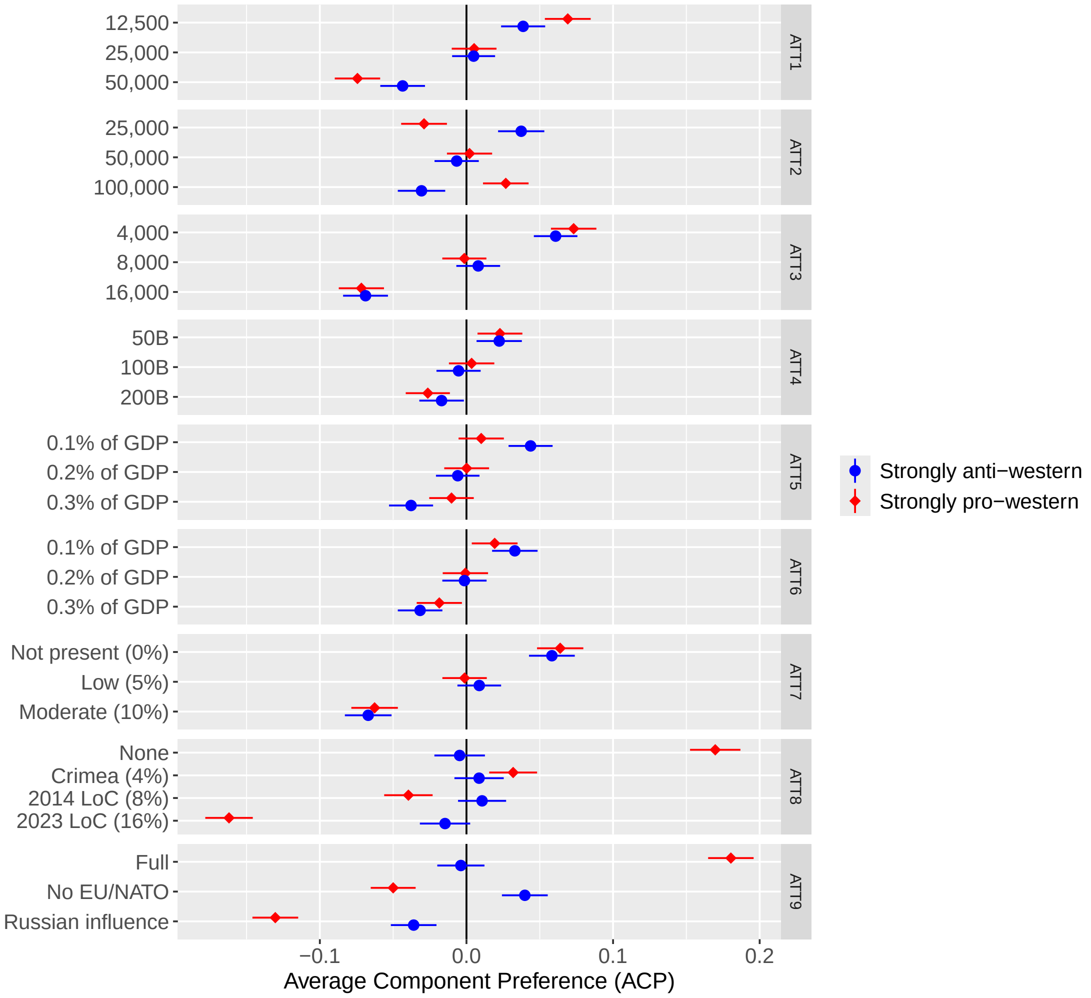

Supplementary Figure 16: Average Component Preferences (ACPs) for the conjoint choice task by subgroups with strong pro-/anti-Western attitudes confirm subgroup heterogeneity on several attributes, substantively relevant particularly for attributes 8 and 9 (political costs). ACPs for the conjoint choice task by the first (strong pro-Western; $N = 18,472$ observations from $N = 2,309$ respondents ($df = 2,308$)) and fourth quartile (strong anti-Western; $N = 18,936$ from $N = 2,367$ respondents ($df = 2,366$)) of the pro/-anti-Western attitudes scale, which is derived by using the first component in PCA. 95% confidence intervals, based on robust standard errors, are shown using a two-tailed test. For description of attributes and reading examples, see Supplementary Figure 15. MMs are presented in Main Figure 4.

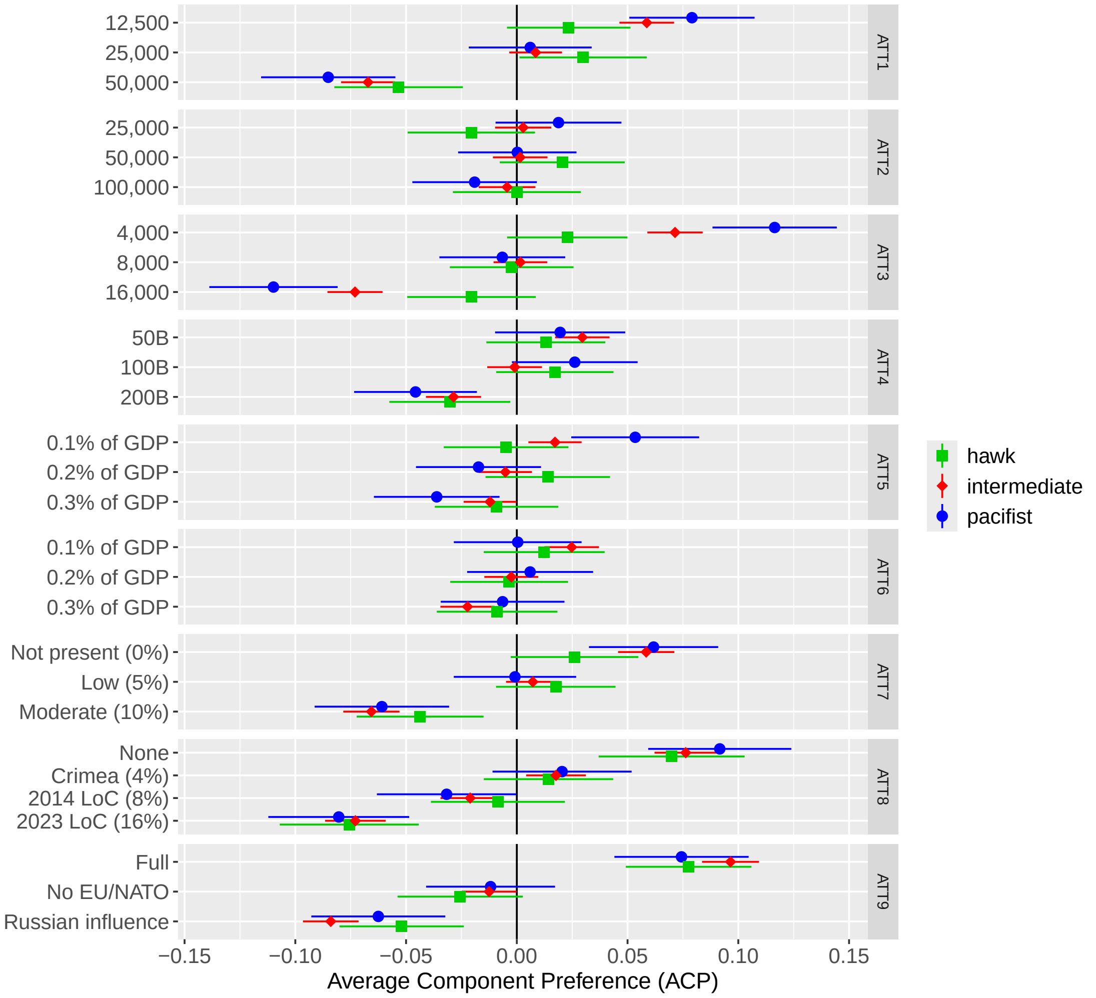

Supplementary Figure 17: Average Component Preferences (ACPs) for the conjoint choice task by hawkish/pacifist respondents indicate subgroup heterogeneity on several attributes. ACPs for the conjoint choice task by pacifistic and hawkish respondents. The former are operationalized by the top quartile of the attitudes toward peace *and* bottom quartile of the attitudes war scale ($N = 5,480$ observations from $N = 685$ respondents ($df = 684$)); the latter by the inverse ($N = 5,616$ observations from $N = 702$ respondents ($df = 701$)). ACPs for intermediate respondents are also shown ($N = 29,040$ observations from $N = 3,630$ respondents ($df = 3,629$)). Attitudes towards war and peace are derived from the first two principal components of the item battery. Top (bottom) quartile has a particularly favorable (unfavorable) view on the relevance of peace (war). Scale inquired for an experimental subset of 50% of all respondents. 95% confidence intervals, based on robust standard errors, are shown using a two-tailed test. For description of attributes and reading examples, see Supplementary Figure 15. MMs are presented in Supplementary Figure 7.

attribute	level	baseline	afcp	se	zstat	pval	conf_high	conf_low
ATT1	25,000	12,500	0.4552	0.00530	-8.4585	<0.001	0.4656	0.4448
ATT1	50,000	12,500	0.4307	0.00540	-12.8513	<0.001	0.4412	0.4201
ATT2	50,000	25,000	0.4983	0.00531	-0.3166	0.752	0.5087	0.4879
ATT2	100000	25,000	0.4942	0.00548	-1.0616	0.288	0.5049	0.4835
ATT3	8,000	4,000	0.4367	0.00531	-11.9303	<0.001	0.4471	0.4263
ATT3	16,000	4,000	0.4244	0.00534	-14.1671	<0.001	0.4349	0.4140
ATT4	100B	50B	0.4799	0.00540	-3.7112	<0.001	0.4905	0.4694
ATT4	200B	50B	0.4696	0.00529	-5.7371	<0.001	0.4800	0.4593
ATT5	0.2% of GDP	0.1% of GDP	0.4794	0.00529	-3.8922	<0.001	0.4898	0.4690
ATT5	0.3% of GDP	0.1% of GDP	0.4766	0.00530	-4.4224	<0.001	0.4870	0.4662
ATT6	0.2% of GDP	0.1% of GDP	0.4724	0.00533	-5.1722	<0.001	0.4829	0.4620
ATT6	0.3% of GDP	0.1% of GDP	0.4774	0.00534	-4.2348	<0.001	0.4878	0.4669
ATT7	Low (5%)	Not present (0%)	0.4507	0.00534	-9.2222	<0.001	0.4612	0.4403
ATT7	Moderate (10%)	Not present (0%)	0.4264	0.00547	-13.4548	<0.001	0.4371	0.4157
ATT8	Crimea (4%)	None	0.4260	0.00716	-10.3333	<0.001	0.4400	0.4120
ATT8	2014 LoC (8%)	None	0.4354	0.00715	-9.0359	<0.001	0.4494	0.4213
ATT8	2023 LoC (16%)	None	0.4107	0.00722	-12.3666	<0.001	0.4249	0.3966
ATT9	No EU/NATO	Full	0.4288	0.00543	-13.1207	<0.001	0.4394	0.4182
ATT9	Russian influence	Full	0.3928	0.00539	-19.8700	<0.001	0.4034	0.3822

Supplementary Table 5: Calculated Average Feature Choice Probabilities (AFCPs) for each attribute level relative to the specified baseline (as explained in Abramson *et al.* [37]) as well as z-statistics and p-values for the two-sided hypothesis test of the null that the AFCP is equivalent to 0.5. Confidence interval = 0.95 and $N = 80,088$ from $N = 10,011$ respondents ($df = 10,010$). The results corroborate the AMCE estimates shown in Main Figure 2. To compare AFCP with AMCE results more easily, one can subtract 0.5 from the AFCP estimates. Interpretation of the first AFCP estimate: Respondents prefer profiles with the feature of 25,000 Ukrainian military casualties 45.5% of the time when it competes against the baseline of 12,500 Ukrainian military casualties. This estimate is statistically different from the null of 0.5 = 50%.

attribute	level_a	level_b	level_c	afcp_ab	afcp_ac	afcp_bc	se_afcp_ab	se_afcp_ac	se_afcp_bc	direct	indirect	wald_stat	wald_p
ATT1	25,000	12,500	50,000	0.4552	0.5569	0.5693	0.00530	0.00530	0.00540	-0.0448	-0.0125	12.6091	<0.001
ATT1	50,000	12,500	25,000	0.4307	0.4431	0.5448	0.00540	0.00530	0.00530	-0.0693	-0.1017	12.6091	<0.001
ATT2	50,000	25,000	100000	0.4983	0.5049	0.5058	0.00531	0.00533	0.00548	-0.0017	-0.0009	0.0076	0.931
ATT2	100000	25,000	50,000	0.4942	0.4951	0.5017	0.00548	0.00533	0.00531	-0.0058	-0.0066	0.0076	0.931
ATT3	8,000	4,000	16,000	0.4367	0.5719	0.5756	0.00531	0.00533	0.00534	-0.0633	-0.0037	42.4236	<0.001
ATT3	16,000	4,000	8,000	0.4244	0.4281	0.5633	0.00534	0.00533	0.00531	-0.0756	-0.1352	42.4236	<0.001
ATT4	100B	50B	200B	0.4799	0.5205	0.5304	0.00540	0.00538	0.00529	-0.0201	-0.0098	1.2007	0.273
ATT4	200B	50B	100B	0.4696	0.4795	0.5201	0.00529	0.00538	0.00540	-0.0304	-0.0406	1.2007	0.273
ATT5	0.2% of GDP	0.1% of GDP	0.3% of GDP	0.4794	0.5096	0.5234	0.00529	0.00530	0.00530	-0.0206	-0.0138	0.5464	0.46
ATT5	0.3% of GDP	0.1% of GDP	0.2% of GDP	0.4766	0.4904	0.5206	0.00530	0.00530	0.00529	-0.0234	-0.0302	0.5464	0.46
ATT6	0.2% of GDP	0.1% of GDP	0.3% of GDP	0.4724	0.5162	0.5226	0.00533	0.00528	0.00534	-0.0276	-0.0065	5.3126	0.021
ATT6	0.3% of GDP	0.1% of GDP	0.2% of GDP	0.4774	0.4838	0.5276	0.00534	0.00528	0.00533	-0.0226	-0.0437	5.3126	0.021
ATT7	Low (5%)	Not present (0%)	Moderate (10%)	0.4507	0.5588	0.5736	0.00534	0.00536	0.00547	-0.0493	-0.0148	14.1103	<0.001
ATT7	Moderate (10%)	Not present (0%)	Low (5%)	0.4264	0.4412	0.5493	0.00547	0.00536	0.00534	-0.0736	-0.1081	14.1103	<0.001
ATT8	Crimea (4%)	None	2014 LoC (8%)	0.4260	0.5491	0.5646	0.00716	0.00705	0.00715	-0.0740	-0.0155	23.4406	<0.001
ATT8	Crimea (4%)	None	2023 LoC (16%)	0.4260	0.5788	0.5893	0.00716	0.00706	0.00722	-0.0740	-0.0105	27.3747	<0.001
ATT8	2014 LoC (8%)	None	2023 LoC (16%)	0.4354	0.5602	0.5893	0.00715	0.00715	0.00722	-0.0646	-0.0291	8.4873	0.004
ATT8	2014 LoC (8%)	None	Crimea (4%)	0.4354	0.4509	0.5740	0.00715	0.00705	0.00716	-0.0646	-0.1231	23.4406	<0.001
ATT8	2023 LoC (16%)	None	2014 LoC (8%)	0.4107	0.4398	0.5646	0.00722	0.00715	0.00715	-0.0893	-0.1249	8.4873	0.004
ATT8	2023 LoC (16%)	None	Crimea (4%)	0.4107	0.4212	0.5740	0.00722	0.00706	0.00716	-0.0893	-0.1528	27.3747	<0.001
ATT9	No EU/NATO	Full	Russian influence	0.4288	0.5426	0.6072	0.00543	0.00541	0.00539	-0.0712	-0.0646	0.5332	0.465
ATT9	Russian influence	Full	No EU/NATO	0.3928	0.4574	0.5712	0.00539	0.00541	0.00543	-0.1072	-0.1138	0.5332	0.465

Supplementary Table 6: Calculated Average Feature Choice Probabilities (AFCPs) for each attribute level relative to every other attribute level, as well as the estimated direct ($AFCP_l(a,b)$-0.5) and indirect ($AFCP_l(a,c)AFCPl(b,c)$) preference measures for each other attribute level c. $N = 80,088$ from $N = 10,011$ respondents ($df = 10,010$). This table, therefore, presents hypothesis tests for detecting violations of transitivity for every triple of levels of the attributes in the conjoint experiment. The p-value is from the hypothesis test where the null hypothesis is the equivalence of the ”direct” and ”indirect” values. Standard errors are cluster-robust and the p-values are from a (two-sided) Wald test. See Abramson *et al.* [37] for further details.

3.5 Statistics Corresponding to Main Figures and Their Interpretation

	(1)<br>choice<br>b/p/ci95
Ukrainian military casualties	
25,000	-0.035<br>(0.000)<br>[-0.044,-0.026]
50,000	-0.084<br>(0.000)<br>[-0.093,-0.074]
Russian military casualties	
50,000	-0.004<br>(0.373)<br>[-0.013,0.005]
100,000	-0.007<br>(0.139)<br>[-0.016,0.002]
Ukrainian civilian casualties	
8,000	-0.042<br>(0.000)<br>[-0.051,-0.034]
16,000	-0.094<br>(0.000)<br>[-0.104,-0.085]
Ukrainian infrastructure loss	
$100B	-0.018<br>(0.000)<br>[-0.027,-0.009]
$200B	-0.032<br>(0.000)<br>[-0.041,-0.023]
[Country] military aid	
0.2% of GDP	-0.017<br>(0.000)<br>[-0.026,-0.008]
0.3% of GDP	-0.026<br>(0.000)<br>[-0.035,-0.017]
[Country] economic aid	
0.2% of GDP	-0.018<br>(0.000)<br>[-0.026,-0.009]
0.3% of GDP	-0.032<br>(0.000)<br>[-0.041,-0.022]
Nuclear strike risk	
Low (5%)	-0.037<br>(0.000)<br>[-0.046,-0.028]
Moderate (10%)	-0.085<br>(0.000)<br>[-0.095,-0.076]
Concessions	
Crimea (4%)	-0.043<br>(0.000)<br>[-0.054,-0.032]
2014 LoC (8%)	-0.066<br>(0.000)<br>[-0.077,-0.055]
2023 LoC (16%)	-0.109<br>(0.000)<br>[-0.120,-0.098]
Sovereignty	
No EU/NATO	-0.066<br>(0.000)<br>[-0.075,-0.056]
Russian influence	-0.110<br>(0.000)<br>[-0.119,-0.100]
Constant	0.790<br>(0.000)<br>[0.773,0.806]
Observations	80088
Degrees of freedom	10010
R-squared	0.032

Supplementary Table 7: Statistics corresponding to results presented in Main Figure 2. Average Marginal Component Effects for the conjoint experimental choice task. Coefficients (b), p-values (p) and 95% confidence intervals (ci95) reported. $N = 80,088$ from $N = 10,011$ respondents ($df = 10,009$). Estimates presented are derived from linear regressions of choice on attribute-level indicators with the theoretically most favorable levels as the baseline using respondent-clustered standard errors.

	(1)	(2)	(3)	(4)	(5)
	DE	IT	UK	FR	US
	b/p/ci95	b/p/ci95	b/p/ci95	b/p/ci95	b/p/ci95
Ukrainian military casualties					
12,500	0.529	0.539	0.552	0.533	0.546
	(0.000)	(0.000)	(0.000)	(0.000)	(0.000)
	[0.517,0.541]	[0.526,0.552]	[0.540,0.563]	[0.520,0.545]	[0.535,0.557]
25,000	0.501	0.503	0.504	0.512	0.503
	(0.000)	(0.000)	(0.000)	(0.000)	(0.000)
	[0.489,0.512]	[0.490,0.517]	[0.493,0.515]	[0.500,0.524]	[0.492,0.514]
50,000	0.470	0.457	0.446	0.457	0.450
	(0.000)	(0.000)	(0.000)	(0.000)	(0.000)
	[0.458,0.482]	[0.445,0.470]	[0.434,0.457]	[0.445,0.469]	[0.439,0.462]
Russian military casualties					
25,000	0.508	0.510	0.492	0.514	0.494
	(0.000)	(0.000)	(0.000)	(0.000)	(0.000)
	[0.496,0.519]	[0.498,0.523]	[0.481,0.504]	[0.502,0.526]	[0.482,0.506]
50,000	0.499	0.506	0.491	0.502	0.501
	(0.000)	(0.000)	(0.000)	(0.000)	(0.000)
	[0.488,0.510]	[0.493,0.518]	[0.480,0.502]	[0.490,0.513]	[0.490,0.513]
100,000	0.493	0.484	0.517	0.485	0.504
	(0.000)	(0.000)	(0.000)	(0.000)	(0.000)
	[0.482,0.505]	[0.471,0.497]	[0.505,0.529]	[0.473,0.497]	[0.493,0.516]
Ukrainian civilian casualties					
4,000	0.538	0.534	0.563	0.544	0.550
	(0.000)	(0.000)	(0.000)	(0.000)	(0.000)
	[0.526,0.550]	[0.521,0.547]	[0.551,0.574]	[0.532,0.556]	[0.539,0.561]
8,000	0.499	0.500	0.511	0.504	0.501
	(0.000)	(0.000)	(0.000)	(0.000)	(0.000)
	[0.487,0.510]	[0.488,0.512]	[0.500,0.522]	[0.493,0.516]	[0.490,0.512]
16,000	0.464	0.465	0.427	0.452	0.449
	(0.000)	(0.000)	(0.000)	(0.000)	(0.000)
	[0.452,0.476]	[0.453,0.478]	[0.416,0.438]	[0.440,0.464]	[0.437,0.460]
Ukrainian infrastructure loss					
$50B	0.517	0.515	0.520	0.514	0.517
	(0.000)	(0.000)	(0.000)	(0.000)	(0.000)
	[0.506,0.529]	[0.502,0.528]	[0.509,0.531]	[0.503,0.526]	[0.506,0.528]
$100B	0.498	0.500	0.497	0.499	0.501
	(0.000)	(0.000)	(0.000)	(0.000)	(0.000)
	[0.487,0.510]	[0.487,0.513]	[0.486,0.508]	[0.487,0.511]	[0.490,0.512]
$200B	0.485	0.485	0.483	0.487	0.482
	(0.000)	(0.000)	(0.000)	(0.000)	(0.000)
	[0.473,0.496]	[0.472,0.498]	[0.472,0.494]	[0.475,0.498]	[0.471,0.493]
[Country] military aid					
0.1% of GDP	0.519	0.512	0.510	0.508	0.520
	(0.000)	(0.000)	(0.000)	(0.000)	(0.000)
	[0.508,0.530]	[0.499,0.525]	[0.499,0.521]	[0.496,0.520]	[0.509,0.531]
0.2% of GDP	0.488	0.507	0.504	0.496	0.491
	(0.000)	(0.000)	(0.000)	(0.000)	(0.000)
	[0.477,0.500]	[0.493,0.520]	[0.493,0.515]	[0.485,0.507]	[0.480,0.502]
0.3% of GDP	0.492	0.481	0.485	0.495	0.489
	(0.000)	(0.000)	(0.000)	(0.000)	(0.000)
	[0.481,0.504]	[0.468,0.494]	[0.474,0.497]	[0.484,0.507]	[0.477,0.500]
[Country] economic aid					
0.1% of GDP	0.519	0.519	0.513	0.506	0.526
	(0.000)	(0.000)	(0.000)	(0.000)	(0.000)
	[0.508,0.530]	[0.506,0.532]	[0.502,0.524]	[0.494,0.517]	[0.515,0.538]
0.2% of GDP	0.498	0.499	0.497	0.498	0.500
	(0.000)	(0.000)	(0.000)	(0.000)	(0.000)
	[0.487,0.509]	[0.487,0.512]	[0.486,0.508]	[0.487,0.510]	[0.488,0.511]
0.3% of GDP	0.483	0.482	0.490	0.496	0.475
	(0.000)	(0.000)	(0.000)	(0.000)	(0.000)
	[0.471,0.495]	[0.468,0.495]	[0.479,0.501]	[0.484,0.507]	[0.464,0.486]
Nuclear strike risk					
Not present (0%)	0.523	0.552	0.557	0.534	0.538
	(0.000)	(0.000)	(0.000)	(0.000)	(0.000)
	[0.511,0.535]	[0.538,0.565]	[0.545,0.569]	[0.522,0.546]	[0.526,0.549]
Low (5%)	0.515	0.497	0.505	0.498	0.505
	(0.000)	(0.000)	(0.000)	(0.000)	(0.000)
	[0.503,0.527]	[0.485,0.510]	[0.494,0.516]	[0.486,0.510]	[0.494,0.516]
Moderate (10%)	0.462	0.452	0.438	0.468	0.457
	(0.000)	(0.000)	(0.000)	(0.000)	(0.000)
	[0.450,0.474]	[0.438,0.465]	[0.426,0.449]	[0.456,0.481]	[0.445,0.469]
Concessions					
None	0.569	0.520	0.571	0.558	0.553
	(0.000)	(0.000)	(0.000)	(0.000)	(0.000)
	[0.555,0.584]	[0.503,0.537]	[0.557,0.584]	[0.543,0.573]	[0.538,0.568]
Crimea (4%)	0.523	0.508	0.517	0.515	0.495
	(0.000)	(0.000)	(0.000)	(0.000)	(0.000)
	[0.509,0.538]	[0.491,0.524]	[0.504,0.531]	[0.501,0.529]	[0.482,0.509]
2014 LoC (8%)	0.477	0.500	0.483	0.485	0.497
	(0.000)	(0.000)	(0.000)	(0.000)	(0.000)
	[0.462,0.492]	[0.483,0.516]	[0.469,0.497]	[0.471,0.500]	[0.484,0.511]
2023 LoC (16%)	0.427	0.474	0.429	0.442	0.456
	(0.000)	(0.000)	(0.000)	(0.000)	(0.000)
	[0.412,0.442]	[0.459,0.489]	[0.415,0.443]	[0.427,0.457]	[0.442,0.470]
Sovereignty					
Full	0.561	0.538	0.592	0.532	0.569
	(0.000)	(0.000)	(0.000)	(0.000)	(0.000)
	[0.549,0.574]	[0.524,0.553]	[0.580,0.604]	[0.520,0.544]	[0.558,0.581]
No EU/NATO	0.503	0.499	0.476	0.502	0.481
	(0.000)	(0.000)	(0.000)	(0.000)	(0.000)
	[0.492,0.515]	[0.486,0.513]	[0.465,0.488]	[0.490,0.514]	[0.470,0.493]
Russian influence	0.436	0.462	0.433	0.466	0.447
	(0.000)	(0.000)	(0.000)	(0.000)	(0.000)
	[0.424,0.448]	[0.450,0.475]	[0.421,0.444]	[0.453,0.478]	[0.436,0.459]
Observations	16024	16008	16016	16016	16024
Degrees of freedom	2002	2000	2001	2001	2002

Supplementary Table 8: Statistics corresponding to results presented in Main Figure 3. Overall $N = 80,088$, $16,008$ to $16,024$ by country (see models). Estimates presented are predictions for support (marginal means) from linear regressions of choice on attribute-level indicators by country subgroup (see model header) using respondent-clustered standard errors. Results with Bonferroni-adjusted confidence intervals are presented in Supplementary Table 9.

	(1)			
	b	ll	ul	p
Ukrainian military casualties				
12,500 × DE	0.5286	0.5111	0.5461	0.0000
12,500 × IT	0.5391	0.5197	0.5586	0.0000
12,500 × UK	0.5520	0.5349	0.5691	0.0000
12,500 × FR	0.5327	0.5138	0.5515	0.0000
12,500 × US	0.5458	0.5288	0.5628	0.0000
25,000 × DE	0.5001	0.4834	0.5169	0.0000
25,000 × IT	0.5033	0.4832	0.5235	0.0000
25,000 × UK	0.5045	0.4879	0.5211	0.0000
25,000 × FR	0.5123	0.4943	0.5302	0.0000
25,000 × US	0.5027	0.4857	0.5197	0.0000
50,000 × DE	0.4698	0.4516	0.4879	0.0000
50,000 × IT	0.4574	0.4385	0.4762	0.0000
50,000 × UK	0.4461	0.4289	0.4634	0.0000
50,000 × FR	0.4567	0.4388	0.4746	0.0000
50,000 × US	0.4500	0.4329	0.4671	0.0000
Russian military casualties				
25,000 × DE	0.5072	0.4898	0.5247	0.0000
25,000 × IT	0.5102	0.4912	0.5292	0.0000
25,000 × UK	0.4930	0.4759	0.5100	0.0000
25,000 × FR	0.5144	0.4967	0.5321	0.0000
25,000 × US	0.4933	0.4759	0.5108	0.0000
50,000 × DE	0.4986	0.4819	0.5152	0.0000
50,000 × IT	0.5055	0.4868	0.5242	0.0000
50,000 × UK	0.4917	0.4755	0.5078	0.0000
50,000 × FR	0.5021	0.4846	0.5195	0.0000
50,000 × US	0.5009	0.4843	0.5175	0.0000
100,000 × DE	0.4926	0.4753	0.5099	0.0000
100,000 × IT	0.4841	0.4646	0.5036	0.0000
100,000 × UK	0.5175	0.4997	0.5353	0.0000
100,000 × FR	0.4853	0.4670	0.5035	0.0000
100,000 × US	0.5039	0.4866	0.5212	0.0000
Ukrainian civilian casualties				
4,000 × DE	0.5374	0.5194	0.5554	0.0000
4,000 × IT	0.5342	0.5154	0.5530	0.0000
4,000 × UK	0.5633	0.5466	0.5799	0.0000
4,000 × FR	0.5442	0.5259	0.5625	0.0000
4,000 × US	0.5495	0.5325	0.5665	0.0000
8,000 × DE	0.4978	0.4812	0.5143	0.0000
8,000 × IT	0.4999	0.4817	0.5182	0.0000
8,000 × UK	0.5117	0.4951	0.5284	0.0000
8,000 × FR	0.5046	0.4877	0.5216	0.0000
8,000 × US	0.5007	0.4844	0.5170	0.0000
16,000 × DE	0.4632	0.4452	0.4811	0.0000
16,000 × IT	0.4654	0.4465	0.4844	0.0000
16,000 × UK	0.4272	0.4107	0.4438	0.0000
16,000 × FR	0.4526	0.4346	0.4707	0.0000
16,000 × US	0.4480	0.4307	0.4653	0.0000
Ukrainian infrastructure loss				
$50B × DE	0.5166	0.4995	0.5336	0.0000
$50B × IT	0.5150	0.4961	0.5338	0.0000
$50B × UK	0.5209	0.5046	0.5372	0.0000
$50B × FR	0.5149	0.4978	0.5320	0.0000
$50B × US	0.5162	0.4997	0.5328	0.0000
$100B × DE	0.4977	0.4806	0.5148	0.0000
$100B × IT	0.5000	0.4800	0.5200	0.0000
$100B × UK	0.4979	0.4810	0.5148	0.0000
$100B × FR	0.4993	0.4813	0.5172	0.0000
$100B × US	0.5004	0.4838	0.5170	0.0000
$200B × DE	0.4841	0.4671	0.5011	0.0000
$200B × IT	0.4847	0.4650	0.5044	0.0000
$200B × UK	0.4835	0.4670	0.5000	0.0000
$200B × FR	0.4874	0.4700	0.5048	0.0000
$200B × US	0.4817	0.4653	0.4980	0.0000
[Country] military aid				
0.1% of GDP × DE	0.5187	0.5023	0.5352	0.0000
0.1% of GDP × IT	0.5122	0.4927	0.5316	0.0000
0.1% of GDP × UK	0.5107	0.4939	0.5275	0.0000
0.1% of GDP × FR	0.5089	0.4910	0.5268	0.0000
0.1% of GDP × US	0.5195	0.5029	0.5360	0.0000
0.2% of GDP × DE	0.4879	0.4710	0.5047	0.0000
0.2% of GDP × IT	0.5066	0.4863	0.5270	0.0000
0.2% of GDP × UK	0.5051	0.4884	0.5218	0.0000
0.2% of GDP × FR	0.4967	0.4801	0.5133	0.0000
0.2% of GDP × US	0.4904	0.4743	0.5066	0.0000
0.3% of GDP × DE	0.4917	0.4743	0.5091	0.0000
0.3% of GDP × IT	0.4805	0.4612	0.4999	0.0000
0.3% of GDP × UK	0.4863	0.4693	0.5032	0.0000
0.3% of GDP × FR	0.4959	0.4785	0.5132	0.0000
0.3% of GDP × US	0.4883	0.4713	0.5052	0.0000
[Country] economic aid				
0.1% of GDP × DE	0.5182	0.5017	0.5348	0.0000
0.1% of GDP × IT	0.5186	0.4992	0.5380	0.0000
0.1% of GDP × UK	0.5137	0.4967	0.5307	0.0000
0.1% of GDP × FR	0.5064	0.4893	0.5236	0.0000
0.1% of GDP × US	0.5255	0.5085	0.5426	0.0000
0.2% of GDP × DE	0.4974	0.4806	0.5143	0.0000
0.2% of GDP × IT	0.4994	0.4803	0.5185	0.0000
0.2% of GDP × UK	0.4980	0.4816	0.5143	0.0000
0.2% of GDP × FR	0.4990	0.4819	0.5160	0.0000
0.2% of GDP × US	0.4987	0.4821	0.5153	0.0000
0.3% of GDP × DE	0.4826	0.4647	0.5006	0.0000
0.3% of GDP × IT	0.4815	0.4615	0.5016	0.0000
0.3% of GDP × UK	0.4906	0.4740	0.5072	0.0000
0.3% of GDP × FR	0.4961	0.4791	0.5132	0.0000
0.3% of GDP × US	0.4739	0.4573	0.4906	0.0000
Nuclear strike risk				
Not present (0%) × DE	0.5225	0.5052	0.5398	0.0000
Not present (0%) × IT	0.5514	0.5315	0.5714	0.0000
Not present (0%) × UK	0.5579	0.5404	0.5753	0.0000
Not present (0%) × FR	0.5344	0.5164	0.5525	0.0000
Not present (0%) × US	0.5371	0.5197	0.5545	0.0000
Low (5%) × DE	0.5144	0.4965	0.5323	0.0000
Low (5%) × IT	0.4969	0.4782	0.5157	0.0000
Low (5%) × UK	0.5058	0.4897	0.5219	0.0000
Low (5%) × FR	0.4984	0.4802	0.5165	0.0000
Low (5%) × US	0.5045	0.4882	0.5207	0.0000
Moderate (10%) × DE	0.4615	0.4441	0.4788	0.0000
Moderate (10%) × IT	0.4513	0.4313	0.4712	0.0000
Moderate (10%) × UK	0.4386	0.4211	0.4561	0.0000
Moderate (10%) × FR	0.4687	0.4505	0.4870	0.0000
Moderate (10%) × US	0.4567	0.4391	0.4743	0.0000
Concessions				
None × DE	0.5699	0.5474	0.5923	0.0000
None × IT	0.5194	0.4929	0.5460	0.0000
None × UK	0.5715	0.5502	0.5927	0.0000
None × FR	0.5586	0.5349	0.5822	0.0000
None × US	0.5522	0.5296	0.5749	0.0000
Crimea (4%) × DE	0.5237	0.5015	0.5459	0.0000
Crimea (4%) × IT	0.5072	0.4812	0.5332	0.0000
Crimea (4%) × UK	0.5180	0.4971	0.5389	0.0000
Crimea (4%) × FR	0.5155	0.4939	0.5370	0.0000
Crimea (4%) × US	0.4944	0.4734	0.5153	0.0000
2014 LoC (8%) × DE	0.4773	0.4546	0.5000	0.0000
2014 LoC (8%) × IT	0.4992	0.4741	0.5243	0.0000
2014 LoC (8%) × UK	0.4838	0.4621	0.5055	0.0000
2014 LoC (8%) × FR	0.4859	0.4639	0.5078	0.0000
2014 LoC (8%) × US	0.4965	0.4758	0.5172	0.0000
2023 LoC (16%) × DE	0.4273	0.4040	0.4505	0.0000
2023 LoC (16%) × IT	0.4737	0.4502	0.4973	0.0000
2023 LoC (16%) × UK	0.4301	0.4083	0.4519	0.0000
2023 LoC (16%) × FR	0.4424	0.4191	0.4656	0.0000
2023 LoC (16%) × US	0.4548	0.4331	0.4765	0.0000
Sovereignty				
Full × DE	0.5605	0.5417	0.5793	0.0000
Full × IT	0.5380	0.5165	0.5595	0.0000
Full × UK	0.5925	0.5749	0.6100	0.0000
Full × FR	0.5327	0.5146	0.5507	0.0000
Full × US	0.5694	0.5525	0.5863	0.0000
No EU/NATO × DE	0.5024	0.4849	0.5198	0.0000
No EU/NATO × IT	0.4993	0.4793	0.5193	0.0000
No EU/NATO × UK	0.4766	0.4598	0.4935	0.0000
No EU/NATO × FR	0.5026	0.4846	0.5206	0.0000
No EU/NATO × US	0.4814	0.4644	0.4984	0.0000
Russian influence × DE	0.4353	0.4174	0.4532	0.0000
Russian influence × IT	0.4621	0.4434	0.4809	0.0000
Russian influence × UK	0.4328	0.4149	0.4506	0.0000
Russian influence × FR	0.4661	0.4474	0.4848	0.0000
Russian influence × US	0.4471	0.4299	0.4643	0.0000
Observations	80088			
Degrees of freedom	10010			

Supplementary Table 9: Statistics corresponding to results presented in Main Figure 3, with coefficients (b), Bonferroni-adjusted 95% confidence intervals (ll as lower, and ul as upper limit) as well as p-values (p). Which margins are different from each other at the 5%-level under Bonferroni-adjustment can directly be seen from Supplementary Table 10. Margins are predicted from a single regression of choice on attribute levels interacted with the country indicator.

	Coefficient	Std. error	Bonferroni groups
Number of Ukrainian soldiers killed? # country			
12,500 # DE	.5286385	.005961	CDEF
12,500 # IT	.5391307	.0066169	EF
12,500 # UK	.551969	.0058213	F
12,500 # FR	.5326812	.0064253	DEF
12,500 # US	.5457935	.0057996	F
25,000 # DE	.500134	.0056942	BC
25,000 # IT	.5033245	.006866	CDE
25,000 # UK	.504509	.005667	CD
25,000 # FR	.5122503	.0061086	CDE
25,000 # US	.5027351	.005787	CD
50,000 # DE	.469772	.0061798	AB
50,000 # IT	.4573818	.0064156	A
50,000 # UK	.4461151	.0058734	A
50,000 # FR	.4566955	.006105	A
50,000 # US	.4499887	.0058228	A
Number of Russian soldiers killed? # country			
25,000 # DE	.5072329	.005944	AB
25,000 # IT	.510205	.0064831	AB
25,000 # UK	.4929659	.0058	AB
25,000 # FR	.514386	.0060253	AB
25,000 # US	.493348	.0059334	AB
50,000 # DE	.4985895	.00567	AB
50,000 # IT	.5054739	.0063708	AB
50,000 # UK	.4916521	.0055092	AB
50,000 # FR	.5020545	.00594	AB
50,000 # US	.5008839	.0056593	AB
100,000 # DE	.4926216	.0059019	AB
100,000 # IT	.4840818	.006633	A
100,000 # UK	.5174789	.0060603	B
100,000 # FR	.4852598	.0062236	A
100,000 # US	.5039045	.0058946	AB
Number of Ukrainian civilians killed? # country			
4,000 # DE	.5373635	.0061274	GH
4,000 # IT	.5341631	.0064017	FGH
4,000 # UK	.563262	.0056772	H
4,000 # FR	.5442108	.0062456	H
4,000 # US	.5494967	.0058053	H
8,000 # DE	.4977717	.0056435	C E
8,000 # IT	.4999436	.006231	DEF
8,000 # UK	.5117323	.0056727	EFG
8,000 # FR	.5046297	.0057822	EF
8,000 # US	.5007041	.0055508	E
16,000 # DE	.4631661	.0061073	BC
16,000 # IT	.4654442	.006459	B D
16,000 # UK	.4272129	.0056329	A
16,000 # FR	.4526187	.0061475	AB
16,000 # US	.4479637	.0058931	AB
Value of destroyed infrastructure in Ukraine? # country			
$50B # DE	.5165583	.0058201	B D
$50B # IT	.5149531	.0064331	CD
$50B # UK	.5209002	.0055614	D
$50B # FR	.5148886	.0058296	CD
$50B # US	.5162373	.0056277	D
$100B # DE	.4977048	.005827	ABCD
$100B # IT	.5000064	.0068028	ABCD
$100B # UK	.4979461	.0057561	ABCD
$100B # FR	.4992662	.0061054	ABCD
$100B # US	.5003834	.0056631	ABCD
$200B # DE	.4841277	.0057941	AB
$200B # IT	.4846978	.0066966	A C
$200B # UK	.4834577	.0056197	A
$200B # FR	.4873894	.0059183	A C
$200B # US	.4816864	.0055725	A
[Country] contribution to military aid to Ukraine? # country			
0.1% of GDP # DE	.5187375	.0056122	B D
0.1% of GDP # IT	.5121534	.0066134	ABC
0.1% of GDP # UK	.5107041	.0057338	ABC
0.1% of GDP # FR	.5088884	.0060936	ABC
0.1% of GDP # US	.5194765	.0056366	CD
0.2% of GDP # DE	.487882	.0057396	AB
0.2% of GDP # IT	.5066142	.0069314	ABC
0.2% of GDP # UK	.5051022	.0056855	ABC
0.2% of GDP # FR	.4967021	.0056589	ABC
0.2% of GDP # US	.4904056	.0055024	A C
0.3% of GDP # DE	.4916988	.0059355	ABC
0.3% of GDP # IT	.4805486	.0066068	A
0.3% of GDP # UK	.4862538	.0057864	A
0.3% of GDP # FR	.4958757	.005912	ABC
0.3% of GDP # US	.4882527	.005771	A C
[Country] contribution to economic aid to Ukraine? # country			
0.1% of GDP # DE	.5182435	.0056282	DEF
0.1% of GDP # IT	.518603	.006615	C EF
0.1% of GDP # UK	.5136869	.0058026	EF
0.1% of GDP # FR	.5064165	.0058377	BCDEF
0.1% of GDP # US	.5255168	.0058046	F
0.2% of GDP # DE	.4974277	.0057302	ABCDEF
0.2% of GDP # IT	.499417	.0065089	ABCDEF
0.2% of GDP # UK	.4979769	.0055764	ABCDEF
0.2% of GDP # FR	.4989524	.0058056	ABCDEF
0.2% of GDP # US	.4987249	.0056573	ABCDEF
0.3% of GDP # DE	.4826461	.0061282	AB D
0.3% of GDP # IT	.4815429	.0068215	ABC
0.3% of GDP # UK	.4905767	.0056596	ABCDE
0.3% of GDP # FR	.4961212	.0058061	ABCDE
0.3% of GDP # US	.4739402	.0056787	A
Risk of a Russian nuclear strike on Ukraine? # country			
Not present (0%) # DE	.5224974	.0058996	DEFG
Not present (0%) # IT	.5514245	.0068035	GH
Not present (0%) # UK	.5578544	.0059332	H
Not present (0%) # FR	.5344498	.0061646	E GH
Not present (0%) # US	.537082	.0059164	FGH
Low (5%) # DE	.5143644	.0060958	DEF
Low (5%) # IT	.4969466	.0063909	CD
Low (5%) # UK	.5058293	.0054889	DE
Low (5%) # FR	.4983581	.0061761	CDE
Low (5%) # US	.504455	.0055251	D F
Moderate (10%) # DE	.4614798	.0059121	AB
Moderate (10%) # IT	.451256	.0067961	AB
Moderate (10%) # UK	.4386117	.0059624	A
Moderate (10%) # FR	.4687182	.0062165	BC
Moderate (10%) # US	.4566986	.0059976	AB
Territorial cessions of Ukraine to Russia? # country			
None # DE	.5698804	.0074189	I
None # IT	.5194129	.0087829	DEFGH
None # UK	.5714723	.0070248	I
None # FR	.5585616	.0078284	HI
None # US	.552236	.0074972	GHI
Crimea (4%) # DE	.5236961	.0073452	FGH
Crimea (4%) # IT	.5071837	.0085999	DEF
Crimea (4%) # UK	.5179926	.0069212	EFG
Crimea (4%) # FR	.515481	.0071246	EFGH
Crimea (4%) # US	.4943682	.0069266	CDEF
2014 LoC (8%) # DE	.4772778	.0075013	BCD
2014 LoC (8%) # IT	.4992011	.0082985	DEF
2014 LoC (8%) # UK	.4837957	.0071738	CDE
2014 LoC (8%) # FR	.4858551	.0072527	BCDE
2014 LoC (8%) # US	.496527	.0068409	DEF
2023 LoC (16%) # DE	.4272556	.0076771	A
2023 LoC (16%) # IT	.4737357	.0077757	BCD
2023 LoC (16%) # UK	.430104	.0072015	A
2023 LoC (16%) # FR	.4423874	.0076914	AB
2023 LoC (16%) # US	.4548277	.007174	ABC
How much political self-determination of Ukraine? # country			
Full # DE	.5604597	.006402	IJ
Full # IT	.5380053	.0073264	G IJ
Full # UK	.5924731	.0059865	K
Full # FR	.5326593	.0061423	HI
Full # US	.5694242	.005756	JK
No EU/NATO # DE	.5023587	.0059449	F
No EU/NATO # IT	.4992869	.0068103	D FG
No EU/NATO # UK	.4766255	.005747	CDEF
No EU/NATO # FR	.5025971	.0061425	EF H
No EU/NATO # US	.4813968	.0058033	BCDEF
Russian influence # DE	.4352929	.006083	A
Russian influence # IT	.4621473	.006376	ABCD
Russian influence # UK	.4327914	.0060825	A
Russian influence # FR	.4661212	.00637	BC E
Russian influence # US	.4471113	.0058493	AB

Supplementary Table 10: Statistics containing coefficient and standard errors (identical to Suppl. Tab. 8 and 9) as well as group comparisons based on Bonferroni-adjusted confidence intervals from Supplementary Table 9. Margins sharing a letter in the group label are not significantly different at the 5% level.

	(1) Strong anti-Western attitudes b/p/ci95	(2) Strong pro-Western attitudes b/p/ci95
Ukrainian military casualties		
12,500	0.5234	0.5516
	(0.0000)	(0.0000)
	[0.5126,0.5343]	[0.5407,0.5625]
25,000	0.5071	0.5025
	(0.0000)	(0.0000)
	[0.4961,0.5181]	[0.4916,0.5134]
50,000	0.4692	0.4466
	(0.0000)	(0.0000)
	[0.4583,0.4801]	[0.4358,0.4574]
Russian military casualties		
25,000	0.5259	0.4855
	(0.0000)	(0.0000)
	[0.5148,0.5370]	[0.4747,0.4963]
50,000	0.4912	0.5004
	(0.0000)	(0.0000)
	[0.4803,0.5021]	[0.4901,0.5106]
100,000	0.4826	0.5137
	(0.0000)	(0.0000)
	[0.4708,0.4944]	[0.5029,0.5245]
Ukrainian civilian casualties		
4,000	0.5417	0.5495
	(0.0000)	(0.0000)
	[0.5309,0.5524]	[0.5385,0.5606]
8,000	0.5071	0.4992
	(0.0000)	(0.0000)
	[0.4964,0.5179]	[0.4887,0.5097]
16,000	0.4518	0.4520
	(0.0000)	(0.0000)
	[0.4411,0.4625]	[0.4409,0.4632]
Ukrainian infrastructure loss		
$50B	0.5160	0.5146
	(0.0000)	(0.0000)
	[0.5051,0.5269]	[0.5042,0.5251]
$100B	0.4975	0.4996
	(0.0000)	(0.0000)
	[0.4866,0.5083]	[0.4883,0.5108]
$200B	0.4868	0.4859
	(0.0000)	(0.0000)
	[0.4762,0.4973]	[0.4752,0.4966]
[Country] military aid		
0.1% of GDP	0.5266	0.5046
	(0.0000)	(0.0000)
	[0.5158,0.5375]	[0.4939,0.5152]
0.2% of GDP	0.4970	0.5006
	(0.0000)	(0.0000)
	[0.4865,0.5075]	[0.4894,0.5118]
0.3% of GDP	0.4760	0.4947
	(0.0000)	(0.0000)
	[0.4651,0.4869]	[0.4833,0.5061]
[Country] economic aid		
0.1% of GDP	0.5178	0.5134
	(0.0000)	(0.0000)
	[0.5072,0.5283]	[0.5026,0.5241]
0.2% of GDP	0.5055	0.5003
	(0.0000)	(0.0000)
	[0.4947,0.5162]	[0.4898,0.5108]
0.3% of GDP	0.4765	0.4862
	(0.0000)	(0.0000)
	[0.4655,0.4874]	[0.4753,0.4972]
Nuclear strike risk		
Not present (0%)	0.5353	0.5465
	(0.0000)	(0.0000)
	[0.5241,0.5464]	[0.5353,0.5577]
Low (5%)	0.5087	0.5012
	(0.0000)	(0.0000)
	[0.4980,0.5194]	[0.4902,0.5121]
Moderate (10%)	0.4563	0.4516
	(0.0000)	(0.0000)
	[0.4451,0.4676]	[0.4403,0.4628]
Concessions		
None	0.4954	0.6242
	(0.0000)	(0.0000)
	[0.4809,0.5098]	[0.6103,0.6380]
Crimea (4%)	0.5020	0.5216
	(0.0000)	(0.0000)
	[0.4883,0.5158]	[0.5082,0.5350]
2014 LoC (8%)	0.5098	0.4756
	(0.0000)	(0.0000)
	[0.4965,0.5231]	[0.4618,0.4895]
2023 LoC (16%)	0.4929	0.3823
	(0.0000)	(0.0000)
	[0.4789,0.5069]	[0.3691,0.3955]
Sovereignty		
Full	0.5001	0.6131
	(0.0000)	(0.0000)
	[0.4887,0.5115]	[0.6007,0.6256]
No EU/NATO	0.5255	0.4700
	(0.0000)	(0.0000)
	[0.5147,0.5364]	[0.4590,0.4811]
Russian influence	0.4743	0.4151
	(0.0000)	(0.0000)
	[0.4634,0.4852]	[0.4036,0.4267]
Observations	18936	18472
Degrees of freedom	2366	2308

Supplementary Table 11: Statistics corresponding to results presented in Main Figure 4. Marginal means for the choice task by subgroups of the first and fourth quartile of pro-/anti-Western attitudes. Reference category is theoretically most favorable level within attribute. $N = 37,408$ overall, with $N = 18,936$ for the subgroup with strong anti- and $N = 18,472$ with strong pro-Western attitudes. Estimates presented (b) are predictions for support from linear regressions of choice on attribute-level indicators within attitude subgroups (see model header) using respondent-clustered standard errors for calculation of p-values (p) and 95% confidence intervals (ci95). Results with Bonferroni-adjusted confidence intervals are presented in Supplementary Table 12.

	(1)			
	b	ll	ul	p
Ukrainian military casualties				
12,500 × prowestern=0	0.5234	0.5089	0.5380	0.0000
12,500 × prowestern=1	0.5508	0.5362	0.5655	0.0000
25,000 × prowestern=0	0.5071	0.4923	0.5219	0.0000
25,000 × prowestern=1	0.5017	0.4871	0.5164	0.0000
50,000 × prowestern=0	0.4692	0.4545	0.4838	0.0000
50,000 × prowestern=1	0.4459	0.4313	0.4604	0.0000
Russian military casualties				
25,000 × prowestern=0	0.5259	0.5110	0.5409	0.0000
25,000 × prowestern=1	0.4851	0.4705	0.4996	0.0000
50,000 × prowestern=0	0.4913	0.4766	0.5060	0.0000
50,000 × prowestern=1	0.4999	0.4861	0.5137	0.0000
100,000 × prowestern=0	0.4827	0.4668	0.4985	0.0000
100,000 × prowestern=1	0.5133	0.4987	0.5278	0.0000
Ukrainian civilian casualties				
4,000 × prowestern=0	0.5416	0.5271	0.5561	0.0000
4,000 × prowestern=1	0.5490	0.5341	0.5639	0.0000
8,000 × prowestern=0	0.5071	0.4926	0.5215	0.0000
8,000 × prowestern=1	0.4986	0.4845	0.5128	0.0000
16,000 × prowestern=0	0.4517	0.4373	0.4661	0.0000
16,000 × prowestern=1	0.4515	0.4365	0.4665	0.0000
Ukrainian infrastructure loss				
$50B × prowestern=0	0.5158	0.5012	0.5305	0.0000
$50B × prowestern=1	0.5141	0.5000	0.5282	0.0000
$100B × prowestern=0	0.4973	0.4828	0.5119	0.0000
$100B × prowestern=1	0.4990	0.4839	0.5142	0.0000
$200B × prowestern=0	0.4866	0.4725	0.5008	0.0000
$200B × prowestern=1	0.4854	0.4710	0.4997	0.0000
[Country] military aid				
0.1% of GDP × prowestern=0	0.5265	0.5119	0.5411	0.0000
0.1% of GDP × prowestern=1	0.5041	0.4897	0.5184	0.0000
0.2% of GDP × prowestern=0	0.4968	0.4827	0.5110	0.0000
0.2% of GDP × prowestern=1	0.5001	0.4850	0.5151	0.0000
0.3% of GDP × prowestern=0	0.4759	0.4612	0.4906	0.0000
0.3% of GDP × prowestern=1	0.4941	0.4788	0.5095	0.0000
[Country] economic aid				
0.1% of GDP × prowestern=0	0.5176	0.5035	0.5318	0.0000
0.1% of GDP × prowestern=1	0.5128	0.4983	0.5273	0.0000
0.2% of GDP × prowestern=0	0.5054	0.4909	0.5199	0.0000
0.2% of GDP × prowestern=1	0.4997	0.4856	0.5138	0.0000
0.3% of GDP × prowestern=0	0.4764	0.4616	0.4911	0.0000
0.3% of GDP × prowestern=1	0.4857	0.4710	0.5004	0.0000
Nuclear strike risk				
Not present (0%) × prowestern=0	0.5350	0.5200	0.5500	0.0000
Not present (0%) × prowestern=1	0.5461	0.5310	0.5612	0.0000
Low (5%) × prowestern=0	0.5084	0.4940	0.5229	0.0000
Low (5%) × prowestern=1	0.5008	0.4861	0.5155	0.0000
Moderate (10%) × prowestern=0	0.4561	0.4409	0.4712	0.0000
Moderate (10%) × prowestern=1	0.4512	0.4361	0.4664	0.0000
Concessions				
None × prowestern=0	0.4952	0.4751	0.5154	0.0000
None × prowestern=1	0.6232	0.6039	0.6426	0.0000
Crimea (4%) × prowestern=0	0.5019	0.4827	0.5211	0.0000
Crimea (4%) × prowestern=1	0.5207	0.5020	0.5394	0.0000
2014 LoC (8%) × prowestern=0	0.5097	0.4911	0.5282	0.0000
2014 LoC (8%) × prowestern=1	0.4747	0.4554	0.4941	0.0000
2023 LoC (16%) × prowestern=0	0.4928	0.4733	0.5124	0.0000
2023 LoC (16%) × prowestern=1	0.3814	0.3629	0.3998	0.0000
Sovereignty				
Full × prowestern=0	0.5000	0.4847	0.5154	0.0000
Full × prowestern=1	0.6135	0.5968	0.6302	0.0000
No EU/NATO × prowestern=0	0.5255	0.5108	0.5401	0.0000
No EU/NATO × prowestern=1	0.4704	0.4555	0.4853	0.0000
Russian influence × prowestern=0	0.4743	0.4596	0.4889	0.0000
Russian influence × prowestern=1	0.4155	0.3999	0.4310	0.0000
Observations	37408			
Degrees of freedom	4675			

Supplementary Table 12: Statistics corresponding to results presented in Main Figure 4, with coefficients (b) Bonferroni-adjusted 95% confidence intervals (ll as lower, and ul as upper limit) as well as p-values (p). Which margins are different from each other can directly be seen from Supplementary Table 13. Margins are predicted from a single regression of choice on attribute levels interacted with the strong pro-/anti-Western attitude subgroup indicator.

	Coefficient	Std. error	Bonferroni groups
Number of Ukrainian soldiers killed? # prowestern			
12,500 # 0	.5234252	.0055159	A
12,500 # 1	.5508451	.0055654	
25,000 # 0	.5070755	.0056054	A
25,000 # 1	.5017399	.0055641	A
50,000 # 0	.4691513	.0055505	
50,000 # 1	.4458507	.0055041	
Number of Russian soldiers killed? # prowestern			
25,000 # 0	.5259317	.0056565	C
25,000 # 1	.4850878	.0055085	A
50,000 # 0	.4912705	.0055737	AB
50,000 # 1	.4999044	.0052378	AB
100,000 # 0	.4826529	.0060045	A
100,000 # 1	.5132799	.005518	BC
Number of Ukrainian civilians killed? # prowestern			
4,000 # 0	.5415998	.005488	C
4,000 # 1	.5489751	.0056366	C
8,000 # 0	.5070508	.0054703	B
8,000 # 1	.4986366	.0053514	B
16,000 # 0	.4517359	.0054511	A
16,000 # 1	.4514869	.0056728	A
Value of destroyed infrastructure in Ukraine? # prowestern			
$50B # 0	.5158433	.0055618	B
$50B # 1	.5141071	.0053334	B
$100B # 0	.4973171	.0055178	AB
$100B # 1	.4990419	.0057364	AB
$200B # 0	.4866282	.0053634	A
$200B # 1	.485369	.005447	A
[Country] contribution to military aid to Ukraine? # prowestern			
0.1% of GDP # 0	.5265074	.0055255	C
0.1% of GDP # 1	.5040585	.0054359	BC
0.2% of GDP # 0	.4968378	.0053624	AB
0.2% of GDP # 1	.5000866	.0057026	B
0.3% of GDP # 0	.4758731	.0055649	A
0.3% of GDP # 1	.4941297	.0058169	AB
[Country] contribution to economic aid to Ukraine? # prowestern			
0.1% of GDP # 0	.5176481	.0053689	C
0.1% of GDP # 1	.512795	.0055018	BC
0.2% of GDP # 0	.5053907	.0054838	BC
0.2% of GDP # 1	.4997261	.0053343	BC
0.3% of GDP # 0	.4763677	.0055884	A
0.3% of GDP # 1	.4856829	.0055758	AB
Risk of a Russian nuclear strike on Ukraine? # prowestern			
Not present (0%) # 0	.5349884	.0056948	CD
Not present (0%) # 1	.5461304	.0057199	D
Low (5%) # 0	.5084335	.0054671	BC
Low (5%) # 1	.5008219	.0055765	B
Moderate (10%) # 0	.4560606	.0057343	A
Moderate (10%) # 1	.4512308	.0057365	A
Territorial cessions of Ukraine to Russia? # prowestern			
None # 0	.4952437	.0073614	AB
None # 1	.6232387	.0070731	
Crimea (4%) # 0	.50191	.0070206	AB
Crimea (4%) # 1	.5206714	.0068339	B
2014 LoC (8%) # 0	.5096676	.0067926	B
2014 LoC (8%) # 1	.474737	.0070654	A
2023 LoC (16%) # 0	.4928064	.0071466	AB
2023 LoC (16%) # 1	.3813772	.0067445	
How much political self-determination of Ukraine? # prowestern			
Full # 0	.500033	.0058172	BC
Full # 1	.6135021	.0063309	
No EU/NATO # 0	.5254575	.0055461	C
No EU/NATO # 1	.4703979	.0056581	A
Russian influence # 0	.4742617	.0055548	AB
Russian influence # 1	.4154785	.0058939	

Supplementary Table 13: Statistics containing coefficients and standard errors (identical to Suppl. Tab. 11 and 12) as well as group comparisons based on Bonferroni-adjusted confidence intervals from Supplementary Table 12. Margins sharing a letter in the group label are not significantly different at the 5% level.

	(1) support b/p/ci95
air defence	3.8593
	(0.0000)
	[3.7634,3.9552]
air defence and tanks	3.9537
	(0.0000)
	[3.8560,4.0513]
air defence, tanks and jets	4.1702
	(0.0000)
	[4.0691,4.2713]
Western soldiers	2.9510
	(0.0000)
	[2.8573,3.0447]
doubling economic aid	3.7752
	(0.0000)
	[3.6784,3.8721]
max. sanctions against Russia	4.6786
	(0.0000)
	[4.5769,4.7802]
Observations	8596
Degrees of freedom	8590

Supplementary Table 14: Statistics corresponding to results presented in Main Figure 6. Marginal means for agreement to provide six different types of military and economic aid to Ukraine based on the split-sample vignette (scale 0 (no) to 7 (full agreement)). P-values from robust standard errors (p) and 95% confidence intervals (ci95) in parentheses. $N = 8,596$. Estimates (b) presented are predictions from linear regressions of aid support on vignette conditions.

	(1) higher Ukrainian winning chances b/p/ci95	(2) higher World War 3 risk b/p/ci95	(3) quicker end to the war b/p/ci95	(4) more Ukrain. suffering/destruction b/p/ci95	(5) more refugees b/p/ci95	(6) domestic econ. disadvantages b/p/ci95
air defense	ref.	ref.	ref.	ref.	ref.	ref.
air def. + tanks	0.1340	0.0788	0.0374	-0.0186	0.0677	0.0368
	(0.0170)	(0.1600)	(0.5097)	(0.7314)	(0.1997)	(0.4985)
	[0.0239,0.2440]	[-0.0311,0.1888]	[-0.0738,0.1486]	[-0.1250,0.0877]	[-0.0357,0.1711]	[-0.0698,0.1435]
air def., tanks + jets	0.4673	0.3002	0.2738	-0.0516	-0.0432	0.0299
	(0.0000)	(0.0000)	(0.0000)	(0.3442)	(0.4213)	(0.5891)
	[0.3553,0.5793]	[0.1907,0.4097]	[0.1622,0.3853]	[-0.1584,0.0553]	[-0.1485,0.0621]	[-0.0785,0.1382]
West. soldiers	0.2778	0.7804	0.1242	0.0125	0.0917	0.3823
	(0.0000)	(0.0000)	(0.0331)	(0.8207)	(0.0897)	(0.0000)
	[0.1662,0.3893]	[0.6685,0.8924]	[0.0100,0.2385]	[-0.0954,0.1203]	[-0.0142,0.1977]	[0.2739,0.4908]
doubling econ. aid	0.3145	0.1000	0.1341	-0.2098	-0.1775	0.3001
	(0.0000)	(0.0716)	(0.0179)	(0.0001)	(0.0008)	(0.0000)
	[0.2037,0.4252]	[-0.0088,0.2089]	[0.0231,0.2451]	[-0.3165,-0.1031]	[-0.2807,-0.0743]	[0.1917,0.4085]
max. Russ. sanctions	0.2097	0.0852	0.2497	-0.2124	0.0158	0.3384
	(0.0002)	(0.1247)	(0.0000)	(0.0001)	(0.7631)	(0.0000)
	[0.1002,0.3193]	[-0.0236,0.1939]	[0.1387,0.3606]	[-0.3165,-0.1082]	[-0.0869,0.1185]	[0.2322,0.4446]
Constant	4.1216	4.1037	3.7058	4.4416	4.4266	4.1384
	(0.0000)	(0.0000)	(0.0000)	(0.0000)	(0.0000)	(0.0000)
	[4.0428,4.2004]	[4.0268,4.1805]	[3.6279,3.7837]	[4.3669,4.5162]	[4.3534,4.4998]	[4.0636,4.2132]
Observations	10011	10011	10011	10011	10011	10011
Degrees of freedom	10005	10005	10005	10005	10005	10005
R-squared	0.008	0.026	0.004	0.004	0.003	0.010

Supplementary Table 15: Statistics corresponding to results presented in Main Figure 7. Coefficients for changes in perceptions of six types of consequences (as indicated in model header) for six different types of Ukraine support (based on the split-sample aid vignette) relative to vignette level 'air defense' as baseline. P-values from robust standard errors (p) and 95% confidence intervals (ci95) in parentheses. $N = 10,011$. Estimates (b) presented are based on separate linear regressions for each dependent variable on vignette condition indicators.

	higher Ukrainian winning chances		higher World War 3 risk		quicker end to the war		more Ukrain. suffering/destruction		more refugees		domestic econ. disadvantages	
	(1)	(2)	(3)	(4)	(5)	(6)	(7)	(8)	(9)	(10)	(11)	(12)
	anti-West.	pro-West.	anti-West.	pro-West.	anti-West.	pro-West.	anti-West.	pro-West.	anti-West.	pro-West.	anti-West.	pro-West.
	b/p/ci95	b/p/ci95	b/p/ci95	b/p/ci95	b/p/ci95	b/p/ci95	b/p/ci95	b/p/ci95	b/p/ci95	b/p/ci95	b/p/ci95	b/p/ci95
air defence	3.7139	4.2404	4.4785	3.7212	3.4253	3.8031	4.4759	4.4348	4.6253	4.3248	4.4203	3.9412
	(0.0000)	(0.0000)	(0.0000)	(0.0000)	(0.0000)	(0.0000)	(0.0000)	(0.0000)	(0.0000)	(0.0000)	(0.0000)	(0.0000)
	[3.5428,3.8850]	[4.0615,4.4193]	[4.3006,4.6564]	[3.5656,3.8769]	[3.2448,3.6058]	[3.6326,3.9735]	[4.3012,4.6507]	[4.2753,4.5942]	[4.4610,4.7896]	[4.1691,4.4805]	[4.2445,4.5960]	[3.7846,4.0977]
air defence and tanks	3.8342	4.5925	4.5520	3.8231	3.5223	3.8204	4.7327	4.3566	4.8861	4.2976	4.6188	3.8123
	(0.0000)	(0.0000)	(0.0000)	(0.0000)	(0.0000)	(0.0000)	(0.0000)	(0.0000)	(0.0000)	(0.0000)	(0.0000)	(0.0000)
	[3.6596,4.0087]	[4.4213,4.7637]	[4.3732,4.7308]	[3.6554,3.9907]	[3.3417,3.7029]	[3.6491,3.9917]	[4.5606,4.9047]	[4.1896,4.5235]	[4.7237,5.0486]	[4.1456,4.4496]	[4.4418,4.7959]	[3.6628,3.9619]
air defence, tanks and jets	3.9103	5.4031	4.8047	3.8648	3.3536	4.4923	4.8549	3.9490	4.8127	3.9949	4.6016	3.6454
	(0.0000)	(0.0000)	(0.0000)	(0.0000)	(0.0000)	(0.0000)	(0.0000)	(0.0000)	(0.0000)	(0.0000)	(0.0000)	(0.0000)
	[3.7281,4.0925]	[5.2500,5.5562]	[4.6244,4.9851]	[3.7119,4.0177]	[3.1640,3.5431]	[4.3349,4.6498]	[4.6756,5.0341]	[3.7919,4.1060]	[4.6393,4.9860]	[3.8371,4.1527]	[4.4155,4.7877]	[3.4927,3.7981]
Western soldiers	3.7475	5.1222	5.1869	4.8409	3.3207	4.3381	4.9091	4.1108	4.9672	4.2358	5.0859	4.1875
	(0.0000)	(0.0000)	(0.0000)	(0.0000)	(0.0000)	(0.0000)	(0.0000)	(0.0000)	(0.0000)	(0.0000)	(0.0000)	(0.0000)
	[3.5684,3.9266]	[4.9615,5.2828]	[5.0064,5.3673]	[4.6614,5.0204]	[3.1293,3.5121]	[4.1547,4.5215]	[4.7354,5.0828]	[3.9397,4.2819]	[4.8012,5.1331]	[4.0678,4.4038]	[4.9123,5.2594]	[4.0204,4.3546]
doubling economic aid	3.7339	5.0274	4.7043	3.7481	3.3091	4.1197	4.5806	3.9401	4.5134	3.9776	4.9220	4.0798
	(0.0000)	(0.0000)	(0.0000)	(0.0000)	(0.0000)	(0.0000)	(0.0000)	(0.0000)	(0.0000)	(0.0000)	(0.0000)	(0.0000)
	[3.5536,3.9142]	[4.8749,5.1800]	[4.5310,4.8776]	[3.5958,3.9004]	[3.1206,3.4976]	[3.9607,4.2787]	[4.4038,4.7575]	[3.7848,4.0955]	[4.3342,4.6927]	[3.8374,4.1177]	[4.7407,5.1034]	[3.9215,4.2381]
max. sanctions against Russia	3.6793	4.9150	4.5582	3.7050	3.4323	4.3525	4.6057	3.8550	4.8337	4.2300	5.0760	4.0750
	(0.0000)	(0.0000)	(0.0000)	(0.0000)	(0.0000)	(0.0000)	(0.0000)	(0.0000)	(0.0000)	(0.0000)	(0.0000)	(0.0000)
	[3.5070,3.8517]	[4.7662,5.0638]	[4.3865,4.7299]	[3.5495,3.8605]	[3.2515,3.6131]	[4.1947,4.5103]	[4.4428,4.7686]	[3.7029,4.0071]	[4.6772,4.9903]	[4.0814,4.3786]	[4.9113,5.2407]	[3.9212,4.2288]
Observations	2367	2309	2367	2309	2367	2309	2367	2309	2367	2309	2367	2309
Degrees of freedom	2361	2303	2361	2303	2361	2303	2361	2303	2361	2303	2361	2303

Supplementary Table 16: Statistics corresponding to results presented in Main Figure 8. Marginal means for the perception of consequences (as indicated in model header) for six different types of military and economic aid to Ukraine based on the split-sample aid vignette by subgroups of the first and fourth quartile of pro-/anti-Western attitudes (as indicated in model header). P-values from robust standard errors (p) and 95% confidence intervals (ci95) in parentheses. $N = 4,676$ overall, with $N = 2,367$ for the subgroup with strong anti- and $N = 2,309$ with strong pro-Western attitudes. Estimates (b) presented are predictions for consequences based on separate linear regressions for each dependent variable on the vignette condition indicators within subgroups as presented in Supplementary Table 17.

	higher Ukrainian winning chances		higher World War 3 risk		quicker end to the war		more Ukrain. suffering/destruction		more refugees		domestic econ. disadvantages	
	(1)	(2)	(3)	(4)	(5)	(6)	(7)	(8)	(9)	(10)	(11)	(12)
	anti-West.	pro-West.	anti-West.	pro-West.	anti-West.	pro-West.	anti-West.	pro-West.	anti-West.	pro-West.	anti-West.	pro-West.
	b/p/ci95	b/p/ci95	b/p/ci95	b/p/ci95	b/p/ci95	b/p/ci95	b/p/ci95	b/p/ci95	b/p/ci95	b/p/ci95	b/p/ci95	b/p/ci95
air defense	ref.	ref.	ref.	ref.	ref.	ref.	ref.	ref.	ref.	ref.	ref.	ref.
air defence and tanks	0.1202	0.3521	0.0735	0.1018	0.0970	0.0173	0.2567	-0.0782	0.2608	-0.0272	0.1986	-0.1288
	(0.3348)	(0.0053)	(0.5678)	(0.3828)	(0.4565)	(0.8883)	(0.0402)	(0.5065)	(0.0269)	(0.8063)	(0.1187)	(0.2433)
	[-0.1242,0.3646]	[0.1044,0.5997]	[-0.1787,0.3257]	[-0.1269,0.3306]	[-0.1584,0.3523]	[-0.2243,0.2590]	[0.0115,0.5020]	[-0.3091,0.1526]	[0.0298,0.4918]	[-0.2449,0.1904]	[-0.0509,0.4480]	[-0.3453,0.0876]
air defence, tanks and jets	0.1964	1.1627	0.3263	0.1436	-0.0718	0.6893	0.3789	-0.4858	0.1873	-0.3299	0.1813	-0.2958
	(0.1235)	(0.0000)	(0.0116)	(0.1971)	(0.5909)	(0.0000)	(0.0030)	(0.0000)	(0.1241)	(0.0036)	(0.1650)	(0.0081)
	[-0.0536,0.4463]	[0.9272,1.3981]	[0.0730,0.5796]	[-0.0746,0.3618]	[-0.3335,0.1900]	[0.4572,0.9213]	[0.1286,0.6293]	[-0.7096,-0.2620]	[-0.0515,0.4262]	[-0.5516,-0.1082]	[-0.0747,0.4373]	[-0.5145,-0.0771]
Western soldiers	0.0336	0.8817	0.7084	1.1197	-0.1046	0.5350	0.4331	-0.3240	0.3419	-0.0890	0.6656	0.2463
	(0.7905)	(0.0000)	(0.0000)	(0.0000)	(0.4356)	(0.0000)	(0.0006)	(0.0066)	(0.0041)	(0.4462)	(0.0000)	(0.0350)
	[-0.2141,0.2812]	[0.6413,1.1222]	[0.4550,0.9618]	[0.8821,1.3572]	[-0.3677,0.1585]	[0.2846,0.7854]	[0.1867,0.6795]	[-0.5579,-0.0901]	[0.1083,0.5754]	[-0.3181,0.1401]	[0.4186,0.9126]	[0.0173,0.4753]
doubling economic aid	0.0199	0.7870	0.2258	0.0269	-0.1162	0.3166	0.1047	-0.4946	-0.1119	-0.3473	0.5018	0.1386
	(0.8750)	(0.0000)	(0.0747)	(0.8086)	(0.3828)	(0.0078)	(0.4091)	(0.0000)	(0.3670)	(0.0012)	(0.0001)	(0.2222)
	[-0.2286,0.2685]	[0.5519,1.0222]	[-0.0225,0.4741]	[-0.1909,0.2447]	[-0.3772,0.1448]	[0.0836,0.5497]	[-0.1440,0.3533]	[-0.7172,-0.2720]	[-0.3550,0.1313]	[-0.5567,-0.1378]	[0.2492,0.7543]	[-0.0840,0.3613]
max. sanctions against Russia	-0.0346	0.6746	0.0797	-0.0162	0.0070	0.5494	0.1298	-0.5798	0.2084	-0.0948	0.6558	0.1338
	(0.7800)	(0.0000)	(0.5273)	(0.8850)	(0.9572)	(0.0000)	(0.2871)	(0.0000)	(0.0718)	(0.3879)	(0.0000)	(0.2319)
	[-0.2774,0.2083]	[0.4419,0.9073]	[-0.1675,0.3270]	[-0.2362,0.2038]	[-0.2485,0.2625]	[0.3172,0.7817]	[-0.1092,0.3687]	[-0.8001,-0.3595]	[-0.0185,0.4353]	[-0.3101,0.1205]	[0.4149,0.8966]	[-0.0856,0.3533]
Constant	3.7139	4.2404	4.4785	3.7212	3.4253	3.8031	4.4759	4.4348	4.6253	4.3248	4.4203	3.9412
	(0.0000)	(0.0000)	(0.0000)	(0.0000)	(0.0000)	(0.0000)	(0.0000)	(0.0000)	(0.0000)	(0.0000)	(0.0000)	(0.0000)
	[3.5428,3.8850]	[4.0615,4.4193]	[4.3006,4.6564]	[3.5656,3.8769]	[3.2448,3.6058]	[3.6326,3.9735]	[4.3012,4.6507]	[4.2753,4.5942]	[4.4610,4.7896]	[4.1691,4.4805]	[4.2445,4.5960]	[3.7846,4.0977]
Observations	2367	2309	2367	2309	2367	2309	2367	2309	2367	2309	2367	2309
Degrees of freedom	2361	2303	2361	2303	2361	2303	2361	2303	2361	2303	2361	2303
R-squared	0.002	0.052	0.017	0.056	0.002	0.025	0.008	0.019	0.008	0.008	0.020	0.013

Supplementary Table 17: Changes in perceived consequences by subgroups of pro-/anti-Western attitudes (i.e., differences in the Marginal Means presented in Supplementary Table 17/Main Figure 8) with air defense as baseline. P-values from robust standard errors (P) and corresponding 95% confidence intervals (ci95) in parentheses. $N = 4,676$ overall, with $N = 2,367$ for the subgroup with strong anti- and $N = 2,309$ with strong pro-Western attitudes. Estimates (b) presented are based on separate linear regressions for each dependent variable on the vignette condition indicators within subgroups.

3.6 Information on Dataset and Software

Description of dataset (descriptive statistics)

Country	N	Age (mean)	Age (median)	Females (N)	Females (%)	Males (N)	Males (%)
Germany	2003	50.13	52	1023	51.07	980	48.93
Italy	2001	50.29	51	1102	55.07	899	44.93
UK	2002	49.53	50	1045	52.20	957	47.80
France	2002	50.73	53	1100	54.95	902	45.05
USA	2003	48.85	50	1062	53.02	941	46.98
Total	10011	49.91	51	5332	53.26	4679	46.74

Supplementary Table 18: Descriptive statistics of our dataset in total and per country showing age (mean and median), as well as gender composition (N and share in percentage).

Software used

All empirical analyses were conducted with Stata 19, using packages [38–42], besides those presented in Supplementary Figures 15-17 and Supplementary Tables 5, 6, and 17, which were calculated with R. For empirical results conducted with R, Supplementary Table 19 presents detailed information on which version of R software [43] and R packages (directly [44–53] and indirectly loaded) were used.

```
R version 4.5.1 (2025-06-13 ucrt)
Platform: x86_64-w64-mingw32/x64
Running under: Windows 11 x64 (build 26100)

Matrix products: default
  LAPACK version 3.12.1

locale:
[1] LC_COLLATE=German_Germany.utf8  LC_CTYPE=German_Germany.utf8    LC_MONETARY=German_Germany.utf8
[4] LC_NUMERIC=C                    LC_TIME=German_Germany.utf8

time zone: Europe/Berlin
tzcode source: internal

attached base packages:
[1] grid      stats     graphics  grDevices utils     datasets  methods   base

other attached packages:
 [1] afcp_0.0.0.9003  remotes_2.5.0    kableExtra_1.4.0 knitr_1.48       cjoint_2.1.1     survey_4.4-2
 [7] survival_3.8-3   Matrix_1.7-4     lmtest_0.9-40    zoo_1.8-12       sandwich_3.1-1   rio_1.2.3
[13] lubridate_1.9.4  forcats_1.0.0    stringr_1.5.2    dplyr_1.1.4      purrr_1.1.0      readr_2.1.5
[19] tidyr_1.3.1      tibble_3.3.0     ggplot2_4.0.0    tidyverse_2.0.0  pacman_0.5.1

loaded via a namespace (and not attached):
 [1] sjlabelled_1.2.0   tidyselect_1.2.1   viridisLite_0.4.2  farver_2.1.2       R.utils_2.12.3
 [6] S7_0.2.0           fastmap_1.2.0      promises_1.3.3     digest_0.6.37      timechange_0.3.0
[11] mime_0.13          lifecycle_1.0.4    magrittr_2.0.4     compiler_4.5.1     rlang_1.1.6
[16] tools_4.5.1        igraph_2.0.3       labeling_0.4.3     curl_7.0.0         xml2_1.4.0
[21] RColorBrewer_1.1-3 withr_3.0.2        R.oo_1.26.0        xtable_1.8-4       future_1.34.0
[26] globals_0.16.3     scales_1.4.0       MASS_7.3-65        insight_1.0.2      cli_3.6.5
[31] crayon_1.5.3       rmarkdown_2.29     ragg_1.2.7         reformulas_0.4.1   generics_0.1.4
[36] rstudioapi_0.17.1  tzdb_0.5.0         minqa_1.2.7        DBI_1.2.3          splines_4.5.1
[41] parallel_4.5.1     mitools_2.4        vctrs_0.6.5        boot_1.3-31        hms_1.1.3
[46] listenv_0.9.1      systemfonts_1.1.0  glue_1.8.0         parallelly_1.38.0  nloptr_2.1.1
[51] codetools_0.2-20   cowplot_1.1.3      stringi_1.8.7      gtable_0.3.6       later_1.4.4
[56] lme4_1.1-37        furrr_0.3.1        pillar_1.11.0      htmltools_0.5.8.1  R6_2.6.1
[61] textshaping_0.3.7  Rdpack_2.6         shiny_1.11.1       evaluate_1.0.3     lattice_0.22-5
[66] haven_2.5.4        R.methodsS3_1.8.2  rbibutils_2.2.16   httpuv_1.6.16      specr_1.0.0
[71] Rcpp_1.1.0         svglite_2.1.3      nlme_3.1-164       xfun_0.53          pkgconfig_2.0.3
```

Supplementary Table 19: Information about version on R Software and R packages used to generate Supplementary Figures 15-17 and Supplementary Tables 5, 6, and 17.

References

1. Trebesch, C. *et al.* The Ukraine Support Tracker: Which Countries Help Ukraine and How? *Kiel Working Papers* **2218.** https://www.kielinstitut.de/publications/the-ukraine-support-tracker-which-countries-help-ukraine-and-how-6540/ (2023).
2. The White House. Remarks by President Biden and President Zelenskyy of Ukraine in Joint Press Conference. https://bidenwhitehouse.archives.gov/briefing-room/speeches-remarks/2022/12/21/remarks-by-president-biden-and-president-zelenskyy-of-ukraine-in-joint-press-conference/ (2022).
3. The White House. Remarks by President Biden on Supporting Ukraine, Defending Democratic Values, and Taking Action to Address Global Challenges | Vilnius, Lithuania. https://bidenwhitehouse.archives.gov/briefing-room/speeches-remarks/2023/07/12/remarks-by-president-biden-on-supporting-ukraine-defending-democratic-values-and-taking-action-to-address-global-challenges-vilnius-lithuania/ (2023).
4. U.S. Department of Defense. U.S. Committed to Stand With Ukraine 'For as Long as It Takes'. https://www.defense.gov/News/News-Stories/Article/Article/3684739/us-committed-to-stand-with-ukraine-for-as-long-as-it-takes/ (2024).
5. German Federal Foreign Office. "For as Long as It Takes": Ukraine's Security Is Also Our Security. https://www.auswaertiges-amt.de/en/aussenpolitik/laenderinformationen/ukraine-node/sicherheitszusagen-ukraine-2644306 (2024).
6. NATO. Statement by the NATO-Ukraine Council Marking Two Years of Russia's Full-Scale Invasion of Ukraine. https://www.nato.int/cps/en/natohq/official_texts_223087.htm (2024).
7. European Parliament. Provide Ukraine with Military Aid for as Long as Necessary, MEPs Say. https://www.europarl.europa.eu/news/en/press-room/20230210IPR74807/provide-ukraine-with-military-aid-for-as-long-as-necessary-meps-say (2023).
8. Chazan, G. Germany's New Far-Left Party Calls for an End to the Ukraine War. *Financial Times.* https://www.ft.com/content/6e78a371-1d95-48f6-a7a9-388d9f2af113 (2024).
9. Sukin, L. & Lanoszka, A. Credibility in Crises: How Patrons Reassure Their Allies. *International Studies Quarterly* **68,** sqae062. doi.org/10.1093/isq/sqae062 (2024).
10. Blankenship, B. & Lin-Greenberg, E. Trivial Tripwires?: Military Capabilities and Alliance Reassurance. *Security Studies* **31,** 92–117. https://doi.org/10.1080/09636412.2022.2038662 (2022).
11. Stolle, D. Aiding Ukraine in the Russian war: unity or new dividing line among Europeans? *European Political Science* **23,** 218–233. https://doi.org/10.1057/s41304-023-00444-7 (2024).
12. Isernia, P., Martini, S. & Cozzi-Fucile, C. Between prudence and selfishness. Pooling the polls on what Italians think of the Ukraine war. *Contemporary Italian Politics* **16,** 340–352. https://doi.org/10.1080/23248823.2024.2341517 (2024).
13. Masch, L. *et al.* Shift in Public Opinion Formations on Defense, Energy, and Migration: The Case of Russia's War Against Ukraine. *International Journal of Public Opinion Research* **35,** edad038. https://doi.org/10.1093/ijpor/edad038 (2023).
14. Mader, M. Increased support for collective defence in times of threat: European public opinion before and after Russia's invasion of Ukraine. *Policy Studies* **45,** 402–422. https://doi.org/10.1080/01442872.2024.2302441 (2024).

15. Wang, C. & Moise, A. D. A unified autonomous Europe? Public opinion of the EU's foreign and security policy. *Journal of European Public Policy* **30,** 1679–1698. https://doi.org/10.1080/13501763.2023.2217230 (2023).

16. Onderco, M., Smetana, M. & Etienne, T. W. Hawks in the making? European public views on nuclear weapons post-Ukraine. *Global Policy* **14,** 305–317. https://doi.org/10.1111/1758-5899.13179 (2023).

17. Mader, M. & Schoen, H. No *Zeitenwende* (yet): Early Assessment of German Public Opinion toward Foreign and Defense Policy After Russia's Invasion of Ukraine. *Politische Vierteljahresschrift* **64,** 525–547. https://doi.org/10.1007/s11615-023-00463-5 (2023).

18. Fernández, Ó., Vandendriessche, M., Saz-Carranza, A., Agell, N. & Franco, J. The impact of Russia's 2022 invasion of Ukraine on public perceptions of EU security and defence integration: a big data analysis. *Journal of European Integration* **45,** 463–485. https://doi.org/10.1080/07036337.2023.2183392 (2023).

19. Forsberg, T. Bottom-up foreign policy? Finland, NATO and public opinion. *Scandinavian Political Studies* **47,** 283–307. https://doi.org/10.1111/1467-9477.12273 (2024).

20. Moise, A. D. & Wang, C. Appeasement or solidarity? Uncovering the drivers of European public opinion on the EU's foreign policy. *European Union Politics* **26,** 418–441. https://doi.org/10.1177/14651165251320837 (2025).

21. Eck, B. & Michel, E. Breaking the stalemate: Europeans' preferences to expand, cut, or sustain support to Ukraine. *Journal of European Public Policy,* 1–28. https://doi.org/10.1080/13501763.2025.2509755 (2025).

22. Pepinsky, T. B., Reiff, Á. & Szabó, K. The Ukrainian Refugee Crisis and the Politics of Public Opinion: Evidence from Hungary. *Perspectives on Politics* **22,** 989–1014. https://doi.org/10.1017/S1537592724000410 (2022).

23. Smeltz, D. & El Baz, L. Americans See High Stakes for Western Security in Russia-Ukraine War. *Chicago Council on Global Affairs.* https://globalaffairs.org/research/public-opinion-survey/americans-see-high-stakes-western-security-russia-ukraine-war (2024).

24. Smeltz, D. & El Baz, L. American Public Support for Assistance to Ukraine Has Waned, But Still Considerable. *Chicago Council on Global Affairs.* https://globalaffairs.org/research/public-opinion-survey/american-public-support-assistance-ukraine-has-waned-still (2023).

25. Pew Research Center. Mixed views on country's level of support to Ukraine in the U.S., Turkey, Hungary and Poland. https://www.pewresearch.org/global/2024/07/02/nato-seen-favorably-in-member-states-confidence-in-zelenskyy-down-in-europe-us/gap_2024-07-02_russia-nato_00_03/ (2024).

26. Hainmueller, J., Hangartner, D. & Yamamoto, T. Validating vignette and conjoint survey experiments against real-world behavior. *Proceedings of the National Academy of Sciences* **112,** 2395–2400. https://doi.org/10.1073/pnas.1416587112 (2015).

27. Horiuchi, Y., Markovich, Z. & Yamamoto, T. Does Conjoint Analysis Mitigate Social Desirability Bias? *Political Analysis* **30,** 535–549. https://doi.org/10.1017/pan.2021.30 (2022).

28. Ferraro, V. Why Russia invaded Ukraine and how wars benefit autocrats: The domestic sources of the Russo-Ukrainian War. *International Political Science Review* **45,** 170–191. https://doi.org/10.1177/01925121231215048 (2024).

29. Kizilova, K. & Norris, P. "Rally around the flag" effects in the Russian–Ukrainian war. *European Political Science* **23,** 234–250. https://doi.org/10.1057/s41304-023-00450-9 (2024).

30. Foa, R. S. & Nezi, R. Piercing the Fog of War: Measuring Russian Public Opinion via Online Search Data. *University of Cambridge, Working Paper.* https://doi.org/10.17863/CAM.96803 (2023).

31. Letki, N., Walentek, D., Dinesen, P. T. & Liebe, U. Has the war in Ukraine changed Europeans' preferences on refugee policy? Evidence from a panel experiment in Germany, Hungary and Poland. *Journal of European Public Policy* **32,** 1–25. https://doi.org/10.1080/13501763.2024.2304610 (2024).

32. Wimmer, A. *et al.* Geo-Political Rivalry and Anti-Immigrant Sentiment: A Conjoint Experiment in 22 Countries. *American Political Science Review* **119,** 1018–1035. https://doi.org/10.1017/S0003055424000753 (2025).

33. Grauvogel, J. *et al.* Public Opinion on Sanctions Compliance and Evasion: Experimental Evidence from the 2022–23 Russia Sanctions. *OSF.* https://doi.org/10.31219/osf.io/7dsx4 (2024).

34. Banerjee, A. *et al.* In Praise of Moderation: Suggestions for the Scope and Use of Pre-Analysis Plans for RCTs in Economics. *National Bureau of Economic Research.* https://www.nber.org/papers/w26993 (2020).

35. Goplerud, M., Imai, K. & Pashley, N. E. Estimating Heterogeneous Causal Effects of High-Dimensional Treatments: Application to Conjoint Analysis. *The Annals of Applied Statistics* **19,** 866–888. https://doi.org/10.1214/24-AOAS1994 (2025).

36. Rudolph, L. Is there a Partisan Divide in Citizens' Preferences on Ukraine Support? Survey-Experimental Evidence from the US. *SocArXiv.* https://doi.org/10.31235/osf.io/5whx9_v1 (2025).

37. Abramson, S. F., Kocak, K., Magazinnik, A. & Strezhnev, A. Aggregation, Interpretation, and Estimation of Preferences in Conjoint Experiments. *SocArXiv.* https://osf.io/preprints/socarxiv/xjre9 (2024).

38. Jann, B. ESTOUT: Stata module to make regression tables (2023).

39. Jann, B. COEFPLOT: Stata module to plot regression coefficients and other results (2023).

40. Jann, B. GRSTYLE: Stata module to customize the overall look of graphs (2020).

41. Jann, B. COLRSPACE: Stata module providing a class-based color management system in Mata (2024).

42. Jann, B. Color palettes for Stata graphics: An update. *The Stata Journal* **23,** 336–385 (2023).

43. R Core Team. R: A Language and Environment for Statistical Computing. https://www.R-project.org/ (2025).

44. Rinker, T. W. & Kurkiewicz, D. pacman: Package Management for R. version 0.5.0. https://cran.r-project.org/web/packages/pacman/index.html (2018).

45. Chan, C.-h., Leeper, T. J., Becker, J. & Schoch, D. rio: A Swiss-army knife for data file I/O. https://cran.r-project.org/package=rio (2023).

46. Csárdi, G. *et al.* remotes: R Package Installation from Remote Repositories, Including 'GitHub'. R package version 2.5.0. https://CRAN.R-project.org/package=remotes (2024).

47. Wickham, H. *et al.* Welcome to the tidyverse. *Journal of Open Source Software* **4,** 1686. https://doi.org/10.21105/joss.01686 (2019).

48. Wickham, H., François, R., Henry, L., Müller, K. & Vaughan, D. dplyr: A Grammar of Data Manipulation. R package version 1.1.4. https://CRAN.R-project.org/package=dplyr (2023).

49. Wickham, H. *ggplot2: Elegant Graphics for Data Analysis* https://ggplot2.tidyverse.org (Springer, New York, 2016).

50. Hainmueller, J., Hopkins, D. & Yamamoto, T. cjoint: Causal Inference in Conjoint Analysis: Understanding Multi-Dimensional Choices via Stated Preference Experiments. *Political Analysis* **22.** R package version 2.1.1, 1–30. https://CRAN.R-project.org/package=cjoint (2014).

51. Strezhnev, A. afcp: Average Feature Choice Probability Estimator for Conjoint Experiments. R package version 0.0.0.9003, commit 34cccb7863d4990368cfb899c7a2e1d98b798816. https://github.com/astrezhnev/afcp (2024).

52. Xie, Y. *knitr: A Comprehensive Tool for Reproducible Research in R* in *Implementing Reproducible Computational Research* (eds Stodden, V., Leisch, F. & Peng, R. D.) ISBN 978-1466561595 (Chapman and Hall/CRC, 2014).

53. Zhu, H. kableExtra: Construct Complex Table with 'kable' and Pipe Syntax. R package version 1.4.0. https://CRAN.R-project.org/package=kableExtra (2024).
