# Supplementary material for: Examining public support for Ukraine’s defense against autocratic aggression
Source: Nat Commun. 2026 Jan 9;17:414. doi: 10.1038/s41467-025-67913-z (PMC12796306; doi:10.1038/s41467-025-67913-z)
Supplement: Supplementary file 2 — Reporting Summary [file 41467_2025_67913_MOESM2_ESM.pdf]

nature portfolio

Corresponding author(s): Lukas Rudolph

Last updated by author(s): Nov 21, 2025

Reporting Summary

Nature Portfolio wishes to improve the reproducibility of the work that we publish. This form provides structure for consistency and transparency in reporting. For further information on Nature Portfolio policies, see our Editorial Policies and the Editorial Policy Checklist.

Statistics

For all statistical analyses, confirm that the following items are present in the figure legend, table legend, main text, or Methods section.

n/a	Confirmed	
☐	☒	The exact sample size (*n*) for each experimental group/condition, given as a discrete number and unit of measurement
☐	☒	A statement on whether measurements were taken from distinct samples or whether the same sample was measured repeatedly
☐	☒	The statistical test(s) used AND whether they are one- or two-sided<br>*Only common tests should be described solely by name; describe more complex techniques in the Methods section.*
☐	☒	A description of all covariates tested
☐	☒	A description of any assumptions or corrections, such as tests of normality and adjustment for multiple comparisons
☐	☒	A full description of the statistical parameters including central tendency (e.g. means) or other basic estimates (e.g. regression coefficient) AND variation (e.g. standard deviation) or associated estimates of uncertainty (e.g. confidence intervals)
☐	☒	For null hypothesis testing, the test statistic (e.g. *F*, *t*, *r*) with confidence intervals, effect sizes, degrees of freedom and *P* value noted<br>*Give P values as exact values whenever suitable.*
☒	☐	For Bayesian analysis, information on the choice of priors and Markov chain Monte Carlo settings
☒	☐	For hierarchical and complex designs, identification of the appropriate level for tests and full reporting of outcomes
☒	☐	Estimates of effect sizes (e.g. Cohen's *d*, Pearson's *r*), indicating how they were calculated

Our web collection on statistics for biologists contains articles on many of the points above.

Software and code

Policy information about availability of computer code

Data collection	No custom code was used to collect data.
Data analysis	Replication codes to reproduce all analyses conducted for this article are available from Harvard Dataverse under https://doi.org/10.7910/DVN/UDBPS1.<br><br>Software used:<br>Stata 19 (including usage of following external packages: gr0073, palettes, colrspace, gr0059_1, st0085_2, mhtreg, moremata);<br>R - version 4.5.1 (using following packages: afcp[0.0.0.9003], remotes[2.5.0], kableExtra[1.4.0], knitr[1.48], cjoint[2.1.1], survey[4.4-2], survival[3.8-3], Matrix[1.7-4], lmtest[0.9-40], zoo[1.8-12], sandwich[3.1-1], rio[1.2.3], lubridate[1.9.4], forcats[1.0.0], stringr[1.5.2], dplyr[1.1.4], purrr[1.1.0], readr[2.1.5], tidyr[1.3.1], tibble[3.3.0], ggplot2[4.0.0], tidyverse[2.0.0], pacman[0.5.1])

For manuscripts utilizing custom algorithms or software that are central to the research but not yet described in published literature, software must be made available to editors and reviewers. We strongly encourage code deposition in a community repository (e.g. GitHub). See the Nature Portfolio guidelines for submitting code & software for further information.

Data

Policy information about availability of data

All manuscripts must include a data availability statement. This statement should provide the following information, where applicable:

- Accession codes, unique identifiers, or web links for publicly available datasets
- A description of any restrictions on data availability
- For clinical datasets or third party data, please ensure that the statement adheres to our policy

The raw data used in this study to reproduce all analyses conducted in this article have been deposited in the Harvard Dataverse database under https://doi.org/10.7910/DVN/UDBPS1. The study design, including the survey instrument, is available in the pre-analysis plan at Open Science Foundation under https://doi.org/10.17605/OSF.IO/TVZSA.

Research involving human participants, their data, or biological material

Policy information about studies with human participants or human data. See also policy information about sex, gender (identity/presentation), and sexual orientation and race, ethnicity and racism.

Reporting on sex and gender	The survey is quota-representative of the distribution of socio-demographics by country, including gender. Hence, the reported findings apply to all genders (see Population characteristics for details). Disaggregated gender data are provided in the individual-level dataset. Gender data were collected by the survey company (YouGov) with the consent of the respondents (self reported, male, female). Since the focus of this study is not to investigate the effect of (socio-economic) demographics on attitudes, no (post-hoc) analyses of gender-differences are included.
Reporting on race, ethnicity, or other socially relevant groupings	The survey is quota-representative of the distribution of socio-demographics by country, including race in the US and, e.g., education in all countries (see Population characteristics for details). The reported findings apply to all these socially relevant groups. Since the focus of this study is not to investigate the effect of (socio-economic) demographics on attitudes, no (post-hoc) analyses of differences are included.
Population characteristics	Respondents were citizens entitled to vote (18+) within each country, quota representative for the official distributions of these characteristics (region, gender, education, age, past voting behavior, political interest (used for UK, French, Italian, and German samples), and country-specific population characteristics; demographics were interlocked). Hence, self-reported gender identity (male or gemale) was used as a quota for the survey data collection to ensure representation by demographic groups as compared to official statistics within each country. Gender was interlocked with age (for France), with age and education (Germany, UK, Italy) and with age, education and race (USA). In the case of the USA the survey company (YouGov) used interlocked quotas for education, race, age and gender (all self-reported by respondents) as well as for interlocked quotas for eduaction and race to ensure a representative US sample. The categories were: "Black", "Hispanic", "Asian, Native American, Two or more races, Other, Middle Eastern" and "White".<br>Furthermore, YouGov included for all countries (interlocked) quotas for educational attainment with country specific (low, middle, high) categories. For the USA four categories ("No HS, High schol graduate", "Some college, 2-year", "4-year" and "Post-grad)" where used.<br>With deviations of maximally about 10% of official distributions, our data directly reflect the underlying populations. YouGov provided weights that bring the sample fully in line with the voting-eligible populations (for the quota scheme).
Recruitment	Respondents were recuited through YouGov drawing on their online access panels. Respondents sign up in YouGovs pool themselves, and are then invited to take part in surveys. Only citizens of the respective country who were at least 18 years old and eligible to vote were surveyed. Individuals participating in the survey were reimbursed by YouGov.
Ethics oversight	Our study has been approved by the Research Ethics Board of the Faculty of Social Sciences, LMU Munich (Study Protocol GZ23-02). The participants gave their informed consent, and all governmental data protection regulations were followed.

Note that full information on the approval of the study protocol must also be provided in the manuscript.

Field-specific reporting

Please select the one below that is the best fit for your research. If you are not sure, read the appropriate sections before making your selection.

☐ Life sciences ☒ Behavioural & social sciences ☐ Ecological, evolutionary & environmental sciences

For a reference copy of the document with all sections, see nature.com/documents/nr-reporting-summary-flat.pdf

Behavioural & social sciences study design

All studies must disclose on these points even when the disclosure is negative.

Study description	Quantitative survey data (individual-level) to identify attitudes regarding Ukraine-support strategies among the mass publics of the US, the UK, Germany, France and Italy. The survey includes survey experiments (a conjoint experiment and a vignette experiment). To analyze the results of these experimental data we mainly use regression based estimates.

Research sample	For our study, we rely on a high-quality quota sample (N ~ 2,000 for each country) from the survey company YouGov's online access panels in the United States, UK, Germany, France, and Italy (citizens entitled to vote (18+) within each country). These countries are the top-5 supporters of Ukraine with respect to combined bi- and mulilateral military and economic aid flows, and central for decision-making in both NATO and EU.<br>Our five country samples mirror the demographic and political distribution (and are therefore quota-representative for each total voting poulation) in age, gender, region, education, past voting behavior, political interest (used for UK, French, Italian, and German samples), and country-specific population characteristics (race and home ownership for the US; urban/rural for France and Germany; the 2019 EU vote for Italy; work sector for France; social grade and the EU referendum vote for the UK). For all five countries combined 53.3% of the respondents were female (and 46.7% male), the mean age is 49.9 and the median age is 51.
Sampling strategy	Only citizens of the respective country who were at least 18 years old and eligible to vote were sampled from YouGov's online panels using national-level political quotas, which map the current distribution of general political preferences in each country. The quota scheme is based on the standard parameters of region, gender, education, and age but goes beyond this by crossing over the above-mentioned criteria and quoting additionally on past voting behavior, political interest, and additional country-specific population characteristics.<br>Sample sizes (n ~ 2,000 per country) where chose to maximize potential number of respondents in each country given the available research budget. Respondents completed 4 round of the conjoint experiment, providing us roughly 8,000 responses per country (and 40,000 in total). This large sample size gives us enough power to detect small effect sizes with reasonable confidence (e.g., for Alpha = 0.05, and Power = 0.8, the minimum detectable effect size for AMCEs of this conjoint experiment is 0.014 for the total sample).
Data collection	The data was collected by YouGov through anonymous online surveys with blinded treatment conditions. No researcher was present during the surveys.
Timing	The survey was in the field from June 14 to August 28, 2023, with almost 90% (N = 8,956) surveyed before July 1.
Data exclusions	After data delivery, we did not exclude data. Exceptions are items with survey non-response (respondent indicating, e.g., don't know), which were list-wise deleted for this specific survey question.<br><br>Prior to data delivery, respondents were excluded by the data collection company as mutually agreed. Respondents were excluded for the following reasons: a) They did not provide consent to participate, b) they did not match required quota cells, c) they have been recognized as straight-liner or speeder during the survey (YouGov has automated, panel-level systems for dealing with straight-liners and persistent speeders in place), d) to match the country populations as closely as possible, YouGov identified a quota-optimized set of ~2000 respondents per country (total N = 10,011) from the original 12,009 replies, which provide the best match to the underlying population distributions used for quotation.
Non-participation	Only valid completes where included in the 12,009 replies total. Respondents which dropped out/declined participation during the survey (conducted on servers of the survey company) were not seen by us.
Randomization	In the conjoint experiment, the levels shown were completly randomized for each profile and each round (uniform randomization without restrictions). The order of attributes was block-randomized, but constant for all four rounds within respondent. The scale direction of the rating task was randomized.<br>For the vignette experiment, respondents were assigned one of six different policy vignettes with equal probability.<br>Of two longer response batteries on foreign policy values and on attitudes towards war/peace, only one was randomly shown to respondents to decrease survey duration.

Reporting for specific materials, systems and methods

We require information from authors about some types of materials, experimental systems and methods used in many studies. Here, indicate whether each material, system or method listed is relevant to your study. If you are not sure if a list item applies to your research, read the appropriate section before selecting a response.

Materials & experimental systems

n/a	Involved in the study
☒	☐ Antibodies
☒	☐ Eukaryotic cell lines
☒	☐ Palaeontology and archaeology
☒	☐ Animals and other organisms
☒	☐ Clinical data
☒	☐ Dual use research of concern
☒	☐ Plants

Methods

n/a	Involved in the study
☒	☐ ChIP-seq
☒	☐ Flow cytometry
☒	☐ MRI-based neuroimaging

Plants

Seed stocks	n/a
Novel plant genotypes	n/a
Authentication	n/a
